# Secondary Metabolites from a Marine-Derived Fungus *Chaetomium globosum* F0831 and Their Anti-Inflammatory Effects

**DOI:** 10.3390/md24100355

**Published:** 2026-10-09

**Authors:** Tuxiang Mo, Xuan Zhang, Chenyang Yi, Hua Wu, Xinrui Xia, Xinjie Ge, Hong Xiao, Wanshan Li, Guangying Chen

**Affiliations:** 1 Key Laboratory of Tropical Medicinal Resource Chemistry of Ministry of Education, Hainan Normal University, Haikou 571158, China; 2 Key Laboratory of Tropical Medicinal Plant Chemistry of Hainan Province, School of Chemistry and Chemical Engineering, Hainan Normal University, Haikou 571158, China; 3 Key Laboratory of Hainan Provincial International Joint Research Center for Comprehensive Utilization and Efficient Conversion of Tropical Medicinal Resources, Hainan Normal University, Haikou 571158, China

**Keywords:** *Chaetomium globosum*, marine-derived fungi, chemical constituents, anti-inflammatory activity, resorcinol derivatives

## Abstract

Four new metabolites, including two resorcinol derivatives, (*S*)-1-(2,4-dihydroxy-3,5-dimethylphenyl)-3-hydroxybutan-1-one (**1**) and 5-[(8*S*)-2-hydroxypropyl]-6-methylbenzene-1,3-diol (**2**), a benzo[c][1,2]dioxine derivative, (*S*)-3,5,7-trimethyl-3,4-dihydrobenzo[c][1,2]dioxine-6,8-diol (**3**), and a cytochalasan alkaloid, 7-O-acetylarmochaetoglobin Q (**5**), along with nine known compounds, were isolated from the marine fungus *Chaetomium globosum* F0831, obtained from a South China Sea marine sediment. Their structures were accomplished by extensive spectroscopic analysis combined with single-crystal X-ray crystallography and ECD calculations (Electronic Circular Dichroism calculations). The anti-inflammatory activity of the isolated compounds was evaluated. Compound **8** was discovered to potentially reduce inflammation through suppression of the AKT/STAT3 pathway, thereby inhibiting the release of inflammatory mediators (iNOS, COX-2) and inflammatory cytokines (IL-6, TNF-*α*, and IL-1*β*).

## 1. Introduction

Marine sediment-derived fungi, an important yet underexplored reservoir for novel bioactive compounds, thrive in extreme environments that promote unique metabolic adaptations [1,2]. The fungus *Chaetomium globosum* is recognized as a prolific source of structurally diverse secondary metabolites with significant biological activities [3]. The compounds produced by this fungus mainly include cytochalasans, azaphilones, indole alkaloids, and anthraquinones [3]. Among these, cytochalasans and azaphilones represent the most abundant and characteristic components of its metabolic repertoire [4,5,6].

Inflammation functions not only as a protective response to injury and infection but also as a unifying mechanism underlying numerous chronic diseases. Among these, systemic chronic inflammation has been recognized as a central driver across the lifespan, contributing to the onset and progression of cardiovascular disease, cancer, and metabolic disorders [7]. Importantly, the transcription factor STAT3 plays a pivotal role in immune regulation and represents a key molecular link bridging chronic inflammation and tumorigenesis [8,9]. Consequently, therapeutic strategies that target STAT3 and related signaling pathways have emerged as promising approaches for the development of novel anti-inflammatory agents [10,11].

**Figure 1:**
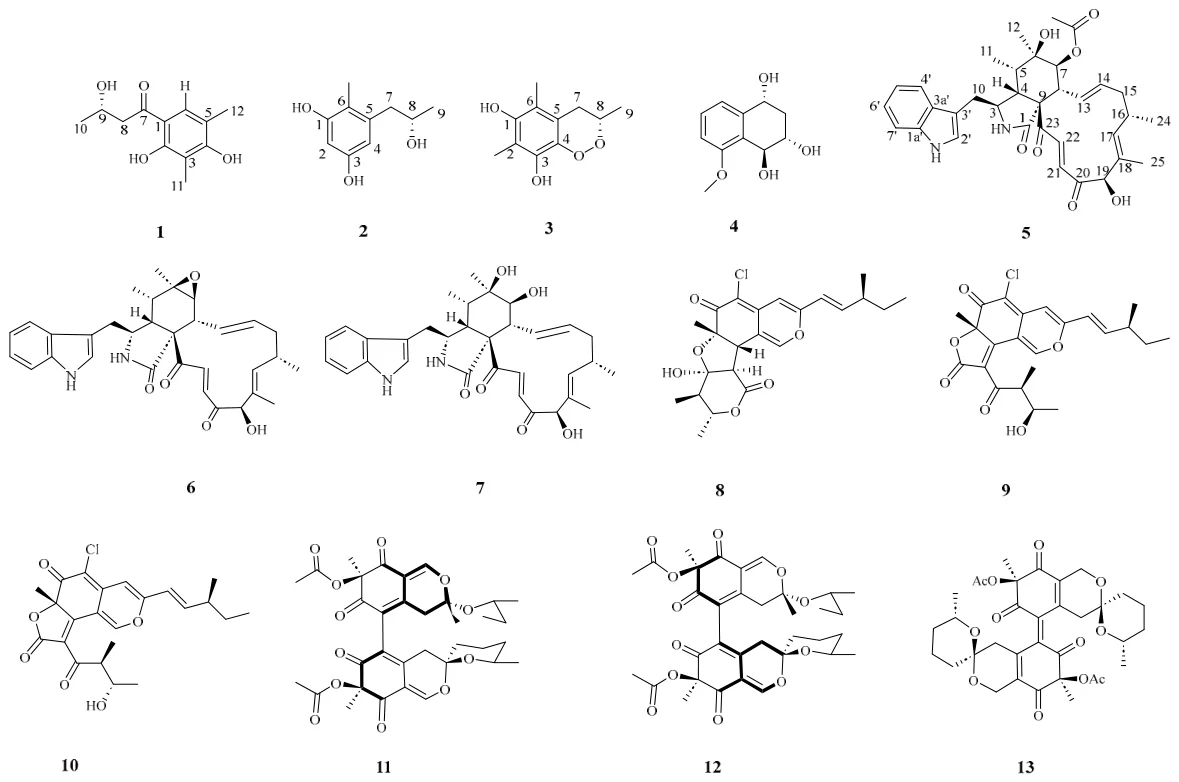
Structures of compounds **1**–**13**.

The marine-derived fungus *Chaetomium globosum* has proven to be a prolific source of structurally diverse polyketides and alkaloids. In the present study, chromatographic investigation of this strain afforded four previously unreported metabolites, encompassing two resorcinol derivatives (**1**, **2**), a rare benzo[c][1,2]dioxine-type endoperoxide (**3**), and a cytochalasan alkaloid (**5**), together with nine known congeners (**4** and **6**–**13**) (Figure 1). More significantly, compound **8** was found to attenuate the inflammatory response, potentially by inhibiting the AKT/STAT3 signaling pathway, thereby blocking the release of inflammatory mediators (iNOS, COX-2) and inflammatory cytokines (IL-6, TNF-*α*, and IL-1*β*). Herein, the detailed isolation, structural elucidation, and bioactivity evaluation of **1**–**13** have been reported.

## 2. Results and Discussion

### 2.1. Chemical Structure Elucidation

**Figure 2:**
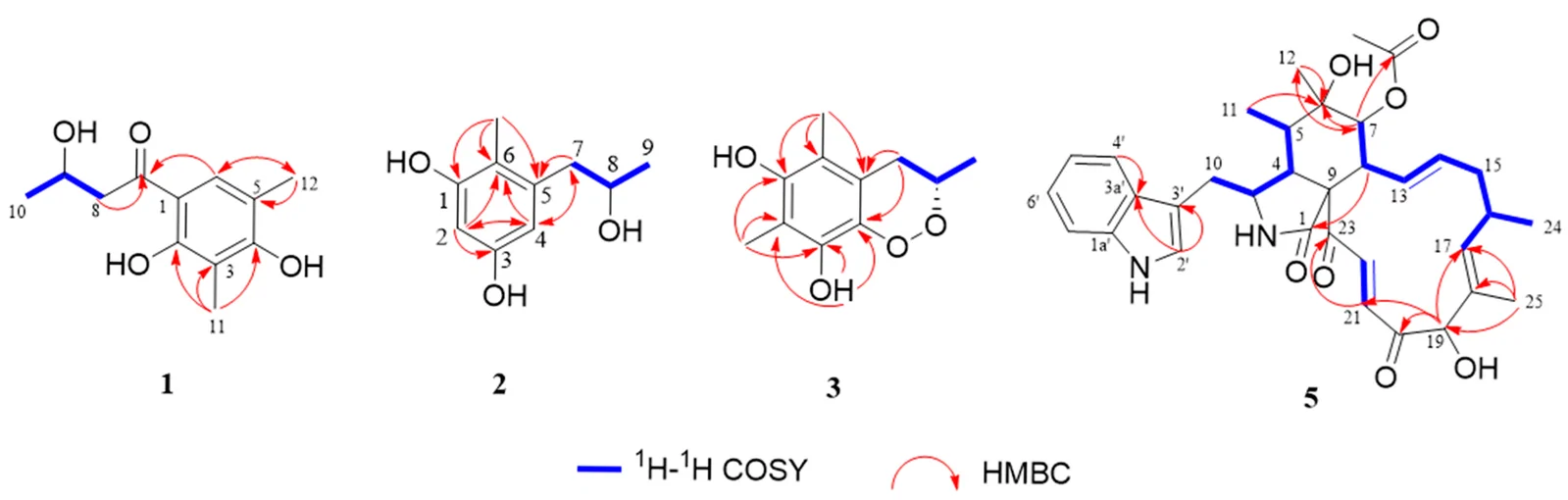
Key ^1^H–^1^H COSY and HMBC correlations of compounds **1**–**3** and **5**.

**Figure 3:**
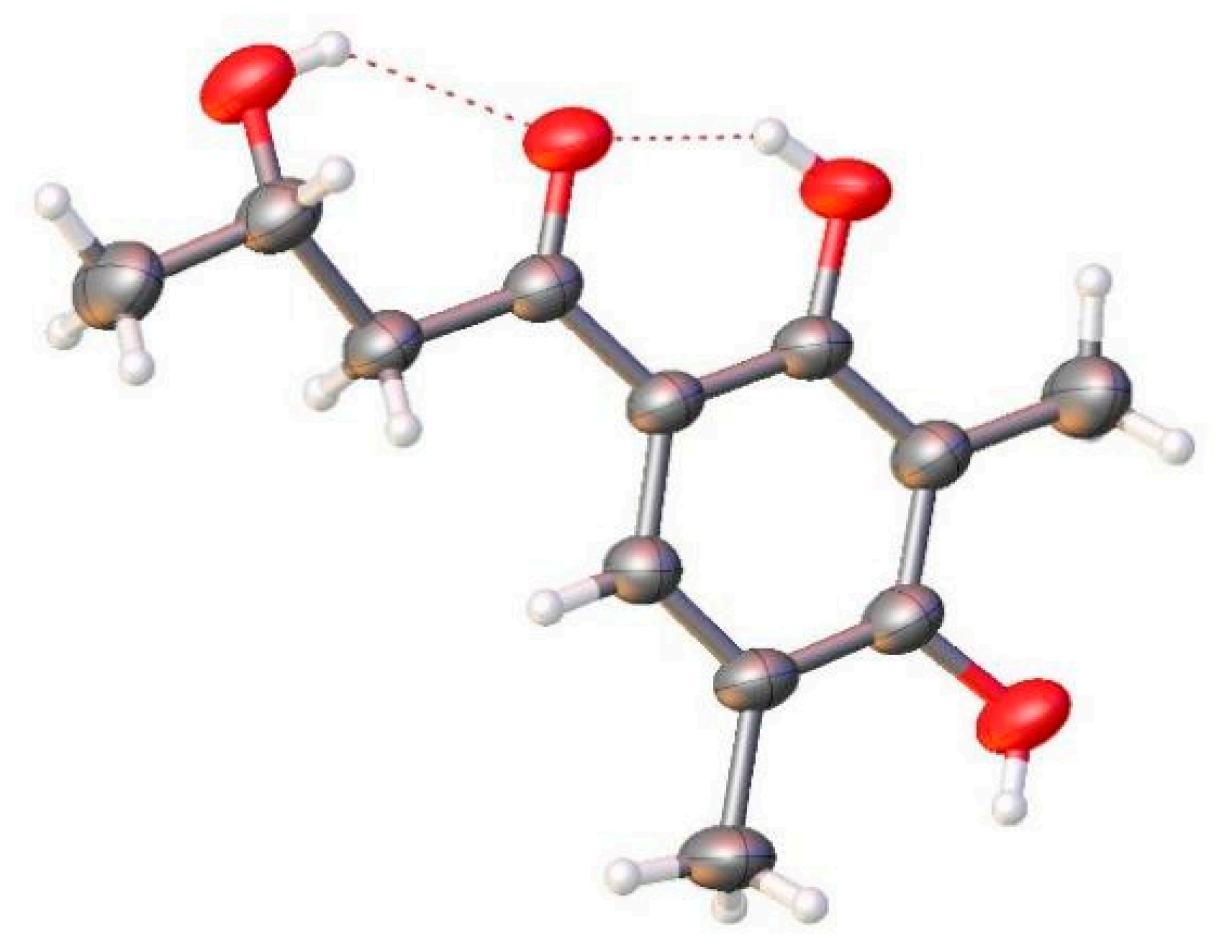
X-ray crystallographic structure of **1**.

(*S*)-1-(2,4-dihydroxy-3,5-dimethylphenyl)-3-hydroxybutan-1-one (**1**) was isolated as needle-like crystals with the molecular formula of C_12_H_16_O_4_ as determined by HRESIMS peak at *m*/*z* 247.0930 [M + Na]^+^ (calcd for C_12_H_16_NaO_4_^+^, 247.0941). The IR (Figure S7) spectrum of **1** suggested the presence of hydroxyl (3443 cm^−1^) and aromatic (1599 cm^−1^) groups. The ^1^H and ^13^C NMR spectra, together with DEPT, showed one carbonyl group, one oxygen-containing methylene group, one methylene group, three methyl groups, and six sp^2^ hybridized tertiary carbons. Detailed analysis of the ^1^H and ^13^C NMR spectra of **1** (Table 1), together with HSQC and HMBC experiments, suggested the presence of a 1,2,3,4-tetrasubstituted butyrophenone. ^1^H–^1^H COSY (Figure 2) correlations were observed between H_3_-10 [*δ*_H_ 1.25 (d, *J* = 6.3 Hz, 3H)] and H-9 [*δ*_H_ 4.33 (m, 1H)], and between H-9 and the diastereotopic methylene protons H_2_-8 [*δ*_H_ 2.92 (dd, *J* = 15.6, 5.0 Hz, 1H) and 3.11 (dd, *J* = 15.6, 7.5 Hz, 1H)], revealing a 3-hydroxybutanoyl moiety. In the HMBC spectrum, cross-peaks from H_2_-8 to the ketone carbonyl carbon C-7 (*δ*_C_ 204.6) and from the aromatic proton H-6 [*δ*_H_ 7.45 (s, 1H)] to C-7 established the attachment of the side chain to C-1 of the aromatic ring. The structure of **1** was further confirmed by comprehensive 2D NMR analyses (Figure 2) and single-crystal X-ray diffraction analyses (Figure 3), which finally established the absolute configuration of **1** as 9*S*. Crystallographic data for compound **1** have been deposited with the Cambridge Crystallographic Data Centre (CCDC 2588332). Thus, we propose that compound **1** is (*S*)-1-(2,4-dihydroxy-3,5-dimethylphenyl)-3-hydroxybutan-1-one.

Compound **2** was obtained as a colorless solid, with a molecular formula of C_10_H_14_O_3_, as deduced from the HRESIMS at *m*/*z* [M-H]^−^ 181.0865 (calcd 181.0870). The ^1^H and ^13^C NMR spectra (Table 1) revealed ten carbon resonances, including one aromatic methyl, one aliphatic methyl, one oxygenated methine, two oxygenated tertiary carbons, one aliphatic methylene, and four tertiary carbons. The downfield carbons at *δ*_C_ 155.8 (C-1) and 154.9 (C-3) indicated oxygenated aromatic carbons adjacent to hydroxyl substituents. The singlet protons at *δ*_H_ 6.13 (H-2, *δ*_C_ 100.3) and *δ*_H_ 6.02 (H-4, *δ*_C_ 108.3) confirmed the presence of a 1,3,5,6-tetrasubstituted benzene ring. The methyl group at *δ*_H_ 1.94 (3H, s, *δ*_C_ 11.0, 6-CH_3_) showed HMBC (Figure 2) correlations to C-1 (*δ*_C_ 155.8), C-5 (*δ*_C_ 139.3) and C-6 (*δ*_C_ 112.7), supporting its attachment at C-6. The ^1^H–^1^H COSY (Figure 2) correlations between H_2_-7/H-8/H_3_-9 confirmed the connectivity of this side chain, revealing a 2-hydroxypropyl structural fragment. HMBC correlations from H-7 to C-4 and C-5 established the linkage of the side chain to the aromatic ring. The optical rotation of 2 was similar to that reported for 5-[2-hydroxypropane-1-yl]-2,6-dimethylbenzene-1,3-diol [12], confirming the C-8 absolute configuration. Thus, compound **2** was elucidated as 5-[(8*S*)-2-hydroxypropyl]- 6-methylbenzene-1,3-diol.

Compound **3** was obtained as a colorless solid, with a molecular formula of C_11_H_14_O_4_ as established by HRESIMS (*m*/*z* [M-H]^−^ 209.0819, calcd for C_11_H_13_O_4_, 209.0819), corresponding to five degrees of unsaturation. Analysis of the ^1^H and ^13^C NMR data indicated that compound **3** is a benzene derivative. The methyl group at *δ*_H_ 1.95 (s, 3H, *δ*_C_ 9.7, 2–CH_3_) showed HMBC correlations (Figure 2) to C-1 (*δ*_C_ 146.1), C-2 (*δ*_C_ 112.2), and C-3 (*δ*_C_ 136.3), supporting its attachment at C-2. The ^1^H–^1^H COSY correlations between H_2_-7/H-8/H_3_-9 confirmed the connectivity of the side chain, revealing a 2-hydroxypropyl fragment. HMBC correlations from H-7 to C-4 and C-5 established the linkage of the side chain to the aromatic ring at C-5. The methyl protons at *δ*_H_ 1.96 (3H, s, *δ*_C_ 12.9) correlated with C-1 (*δ*_C_ 146.1), C-5 (*δ*_C_ 123.2), and C-6 (*δ*_C_ 111.5), establishing the location of the methyl group at C-6. The 3-OH proton signal at *δ*_H_ 8.23 (1H, s) correlated with C-2 (*δ*_C_ 112.2), C-3 (*δ*_C_ 136.3), and C-4 (*δ*_C_ 139.9), confirming the placement of the hydroxy group at C-3. The remaining unsaturation number, combined with high-resolution mass spectrometry data, suggested that a peroxide ring was likely present in compound **3**. The IR (Figure S22) spectrum of compound **3** exhibited a sharp absorption band at 823 cm^−1^, assigned to the stretching vibration of the peroxide bond (O–O); together with the spectroscopic data of bombaxoin, which shares the same peroxide-containing 3-methyl-3,4-dihydrobenzo[c][1,2]dioxine skeleton [13], this established the planar structure of compound 3. To establish the absolute configuration of compound **3**, ECD calculations were performed. The calculated ECD spectrum of (*S*)-**3a** was in good agreement with the experimental CD spectrum, allowing the absolute configuration of compound **3** to be assigned as (*S*). Compound **3** was thus elucidated as (*S*)-3,5,7-trimethyl-3,4-dihydrobenzo[c][1,2]dioxine-6,8-diol.

7-O-acetylarmochaetoglobin Q (**5**) was isolated as a brown oily substance with the molecular formula of C_34_H_40_N_2_O_7_ determined by HRESIMS peak at *m*/*z* 587.2770 [M-H]—(calcd for C_34_H_39_N_2_O_7_, 587.2762). The ^1^H and ^13^C NMR data of compound **5** (Table 2) were closely similar to those of chaetoglobosin Q (**7**) [14], indicating the same cytochalasin skeleton. The remarkable difference between compound **5** and chaetoglobosin Q was that the hydroxyl group at C-7 was replaced by an acetyl group, which was verified by HMBC correlation from H-7 to 7-OCOCH_3_ (Figure 2). The structure of **5** was finally elucidated by the analysis of the HSQC, COSY, and HMBC experimental data.

**Figure 4:**
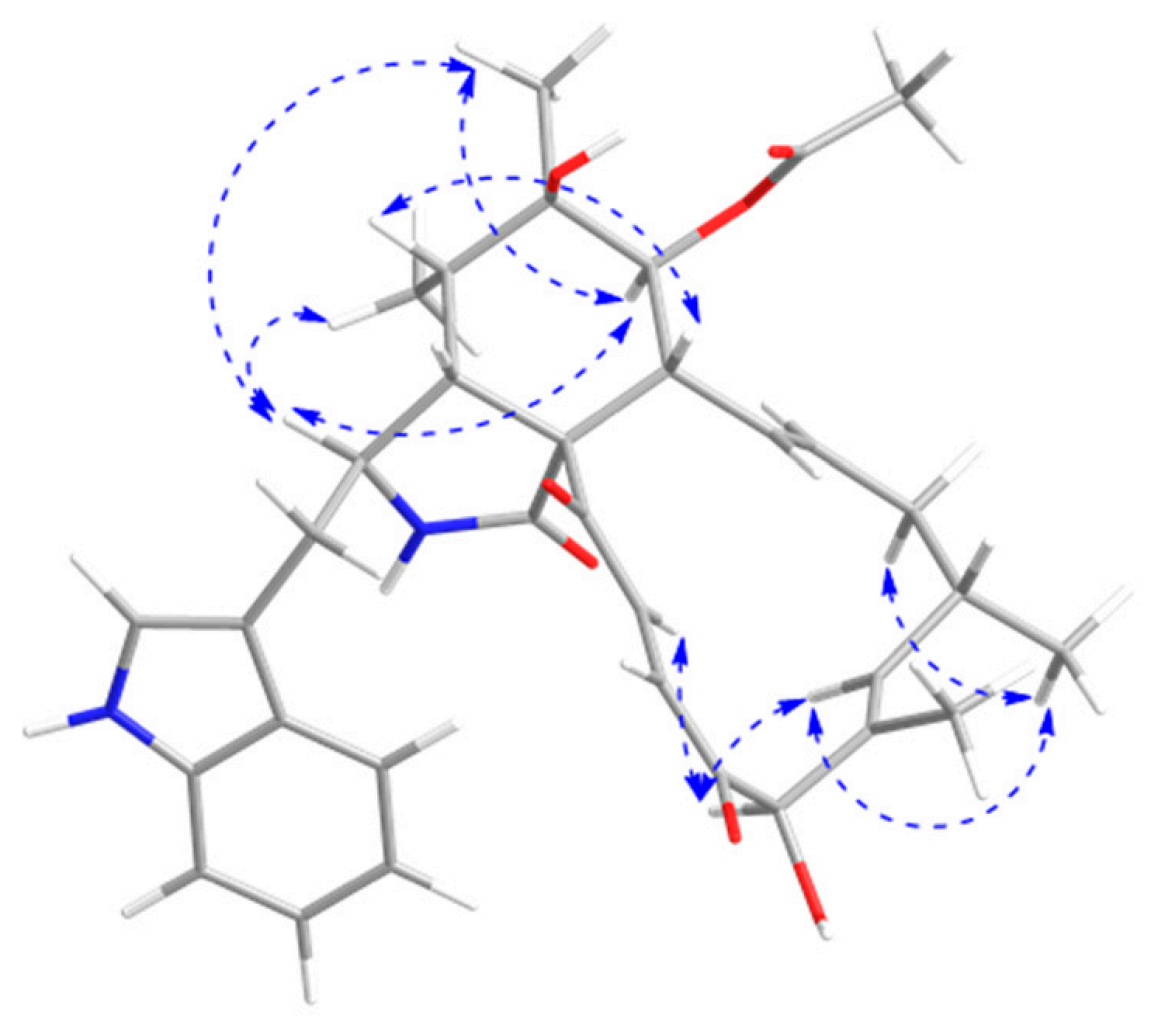
Key NOESY correlations of compound **5**.

The relative stereochemistry of compound **5** was assigned through a combination of NOESY data (Figure 4) and analysis of vicinal coupling constants. The correlations between 11-Me/H-3, H-4/H-5, H-4/H2-10, H-5/H-6, and H-6/H-8 indicated the *α*-orientations of H-3, 11-Me, and 12-Me, and the *β*-orientations of H-4, H-5, H-6, H-8, and H_2_-10 in the perhydro-isoindolone ring. The correlations from H-8 to H-14, from H-14 to H-16, from H-13 and H-17 to 15a, and from H-17 to H-19 and H-22 implied that 16-Me and H-19 were *α*-configured. The correlations between H-17 and H-19 and the large coupling constants of H-21 and H-22 (*J* = 16.7 Hz) and H-13 and H-14 (*J* = 14.7 Hz) confirmed these olefinic protons were trans-configured, and this was consistent with the relative configuration of the analogue chaetoglobosin A (**6**), which possesses the same macrocyclic ring as that of **5**. The absolute configuration of chaetoglobosin A (**6**) was determined by X-ray diffraction [15]. Biosynthetically, compound **5** is derived from chaetoglobosin A (**6**) through post-modification reactions. Thus, the absolute configuration of **5** was postulated to be the same as that of **6**.

**Figure 5:**
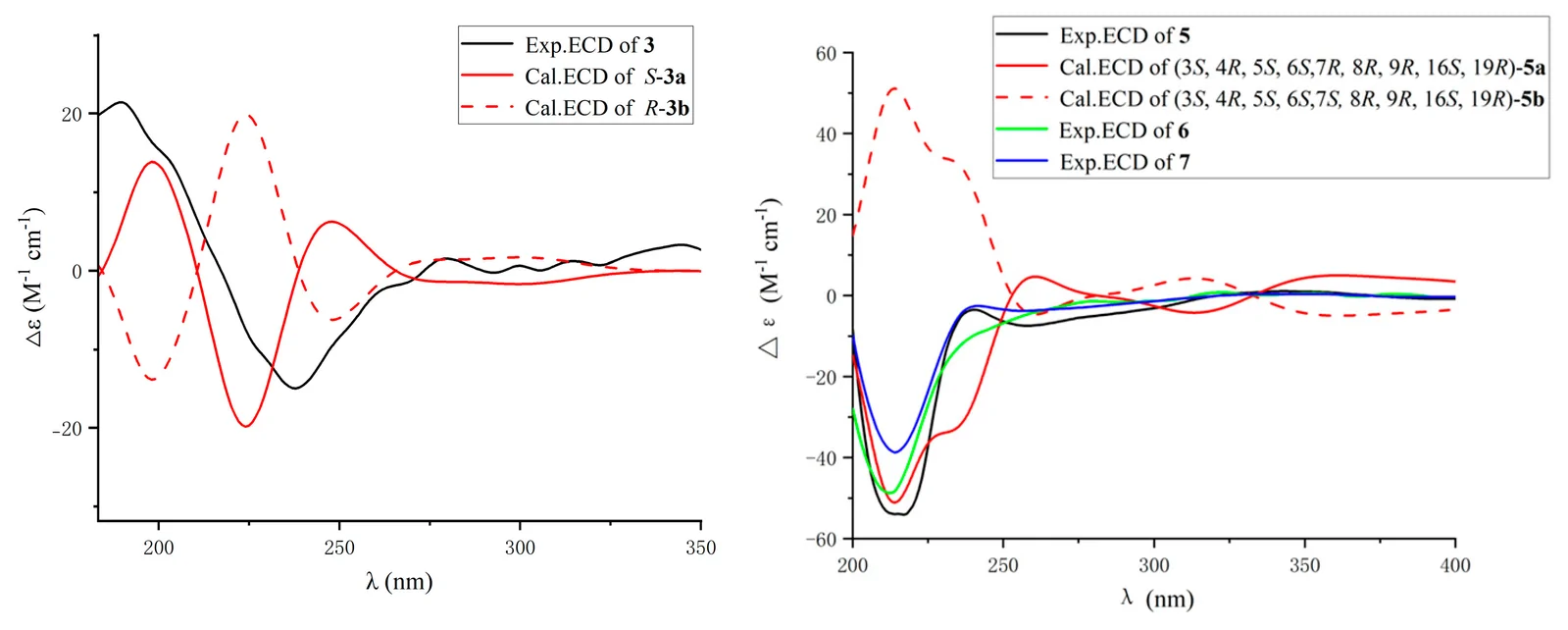
Experimental and calculated ECD spectra of **3** and **5**–**7**.

To support the above-mentioned conclusion, the absolute configuration of compound **5** was unambiguously determined by comparing its experimental ECD spectrum with those calculated via TDDFT, following the established protocols [16,17]. The calculated ECD spectrum of the (3*S*, 4*R*, 5*S*, 6*S*, 8*R*, 9*R*, 16*S*, 19*R*)-isomer matched well with the experimental ECD spectrum of compound **5** (Figure 5). In addition, compounds **5**–**7** possess the same stereogenic centers, and the CD spectra of compounds **6** and **7** were consistent with that of compound **5**, further supporting that compounds **5**–**7** share the same absolute configuration. Therefore, the stereochemistry of compound **5** was determined to be 3*S*, 4*R*, 5*S*, 6*S*, 8*R*, 9*R*, 16*S*, and 19*R*, which is consistent with its biogenetic origin.

Compounds **4** and **6**–**13** were characterized as follows: Xylariol A (**4**) [18], Chaetoglobosin A (**6**) [14], Chaetoglobosin Q (**7**) [19], Chaetomugilin D (**8**) [20], Chaetoviridin A (**9**) [21], 5′-epichaetoviridin A (**10**) [22], Cochliodone A (**11**) [23], Cochliodone B (**12**) [23], and Thielavialide E (**13**) [24]. Their identification was based on comparison of their ^1^H NMR and ^13^C NMR spectra with those reported in the literature.

### 2.2. Biological Activity

The inhibitory effects of all isolated compounds (**1**–**13**) on NO production were evaluated in LPS-induced RAW 264.7 macrophages using dexamethasone as a positive control. Cell viability was determined by MTT assay to confirm whether the observed inhibition of NO production was attributable to cytotoxicity. The results showed that all compounds exhibited no or weak cytotoxic effects (cell viability > 90%) on RAW 264.7 cells at a concentration of 40 μM. Compounds **8** and **9** demonstrated inhibitory effects on LPS-induced inflammatory responses. Compound **8** exhibited the lowest IC_50_ value among the isolated compounds (10.99 ± 0.17 μM) in the NO inhibition assay, while compound **9** showed an IC_50_ value of 28.56 ± 0.39 μM; the positive control dexamethasone showed an IC_50_ value of 35.44 ± 2.27 μM under the tested conditions.

Inflammation plays an important role in the development and prognosis of various diseases. In this study, we evaluated the anti-inflammatory activity of compound **8** in an LPS-induced inflammation model.

**Figure 6:**
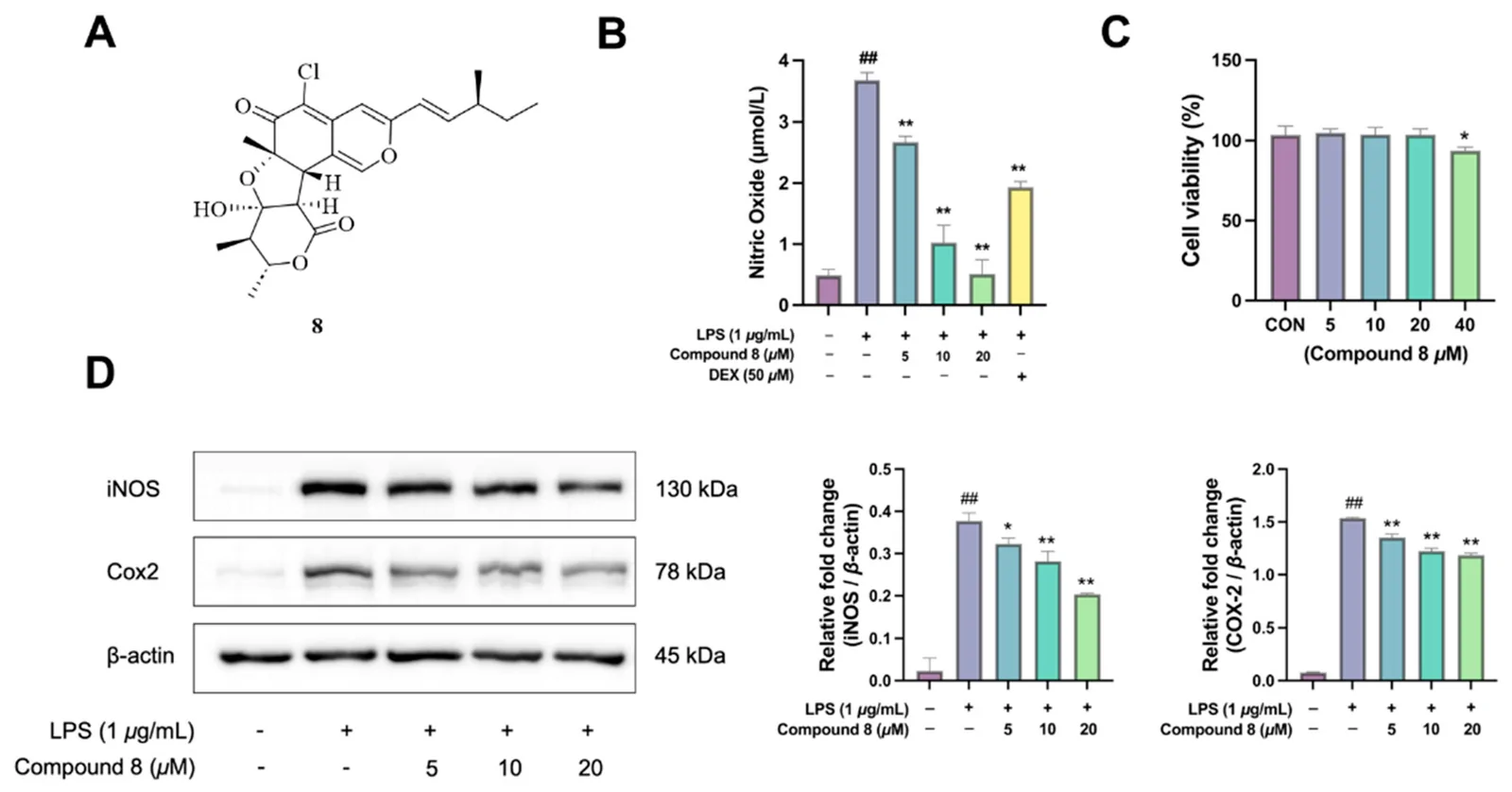
Pretreatment with compound **8** attenuated LPS-mediated inflammatory outcomes in RAW264.7 cells. (**A**) Chemical structure of compound **8**. (**B**) Cell culture supernatants were collected and NO concentrations determined by a commercial kit. (**C**) Cell viability of compound **8** at doses of 5, 10, 20, and 40 μM. (**D**) Cell lysates were obtained and used to determine COX-2, iNOS, and β-actin concentrations by Western blotting. ## *p* < 0.01 vs. Con, * *p* < 0.05, ** *p* < 0.01 vs. LPS. *n* ≥ 3.

NO, a critical pro-inflammatory mediator, serves as a signaling molecule in the induction of inflammation. Under pathological conditions, NO is primarily synthesized by the iNOS enzyme and is subsequently oxidized to the cytotoxic peroxynitrite [25]. Both iNOS and COX-2 play pivotal roles in the inflammatory response: iNOS produces NO, while COX-2 facilitates the production of pro-inflammatory prostaglandins [26]. In this study, pretreatment with compound **8** significantly suppressed NO production at non-cytotoxic concentrations (Figure 6). Western blot analysis further revealed that compound **8** downregulated iNOS protein levels in LPS-treated RAW264.7 cells. Additionally, we observed that compound **8** sharply reduced the protein levels of COX-2. In conclusion, compound **8** mitigates LPS-induced inflammation by inhibiting the overproduction of NO through the downregulation of both COX-2 and iNOS protein levels.

**Figure 7:**
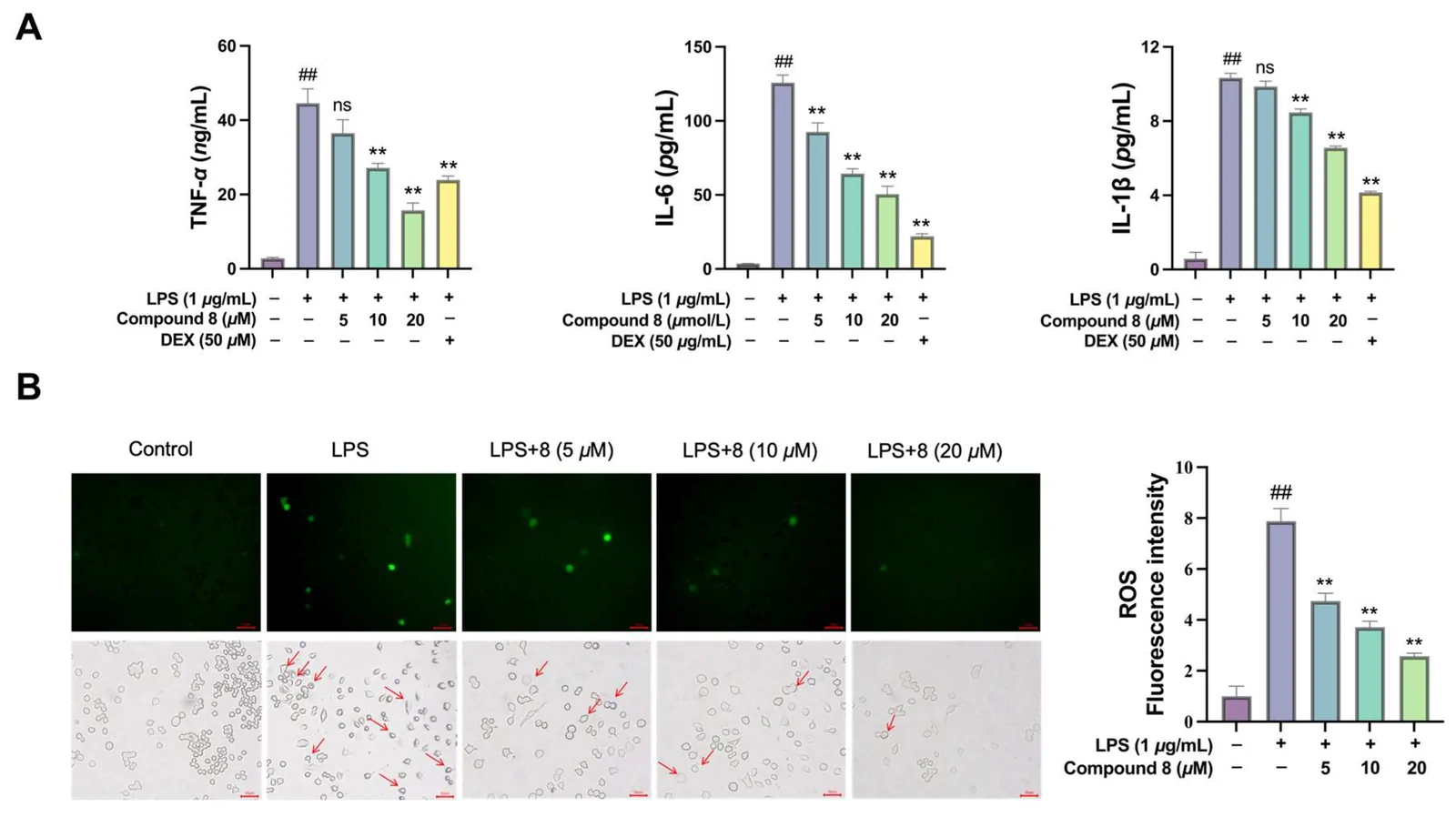
Impact of compound **8** on cytokine secretion and ROS release in LPS-treated RAW264.7 cells. Cell pre-treatment was done for 1 h using different compound **8** concentrations (5, 10 and 20 μM) followed by LPS (1 μg/mL) treatment for 24 h. (**A**) Supernatants of the cell cultures were obtained and used to determine IL-1β and IL-6 as well as TNF-α concentrations by ELISA. (**B**) After treating cells with DCFH-DA fluorescent probe, ROS levels were detected by fluorescence microscopy. Red arrows indicate inflammatory cells with ROS accumulation. Scale bar, 50 µm. ns: not significant; ## *p* < 0.01 vs. Con, ** *p* < 0.01 vs. LPS. *n* ≥ 3.

Activated inflammatory cells produce high amounts of ROS, as well as inflammatory cytokines. Excessive ROS is associated with cell damage and apoptosis; ROS can also synergistically act with NO to enhance oxidative stress and damage [27]. In compound **8**-pre-treated LPS-treated RAW264.7 cells, we observed a reduced green fluorescence intensity, implying that excessive ROS was effectively scavenged (Figure 7). Cytokines mediate cell–cell communication. Excessive IL-6, TNF-*α*, and IL-1*β* promote inflammation, leading to pain, fever, and increased permeability. While these cytokines are protective at normal levels, their overproduction drives inflammatory diseases, making their inhibition crucial for controlling inflammation [28]. We found that pretreatment with compound **8** significantly suppressed pro-inflammatory factor levels, including those of IL-6, TNF-α, and IL-1β, implying that it exhibits good anti-inflammation effects against LPS-treated RAW 264.7 cells.

**Figure 8:**
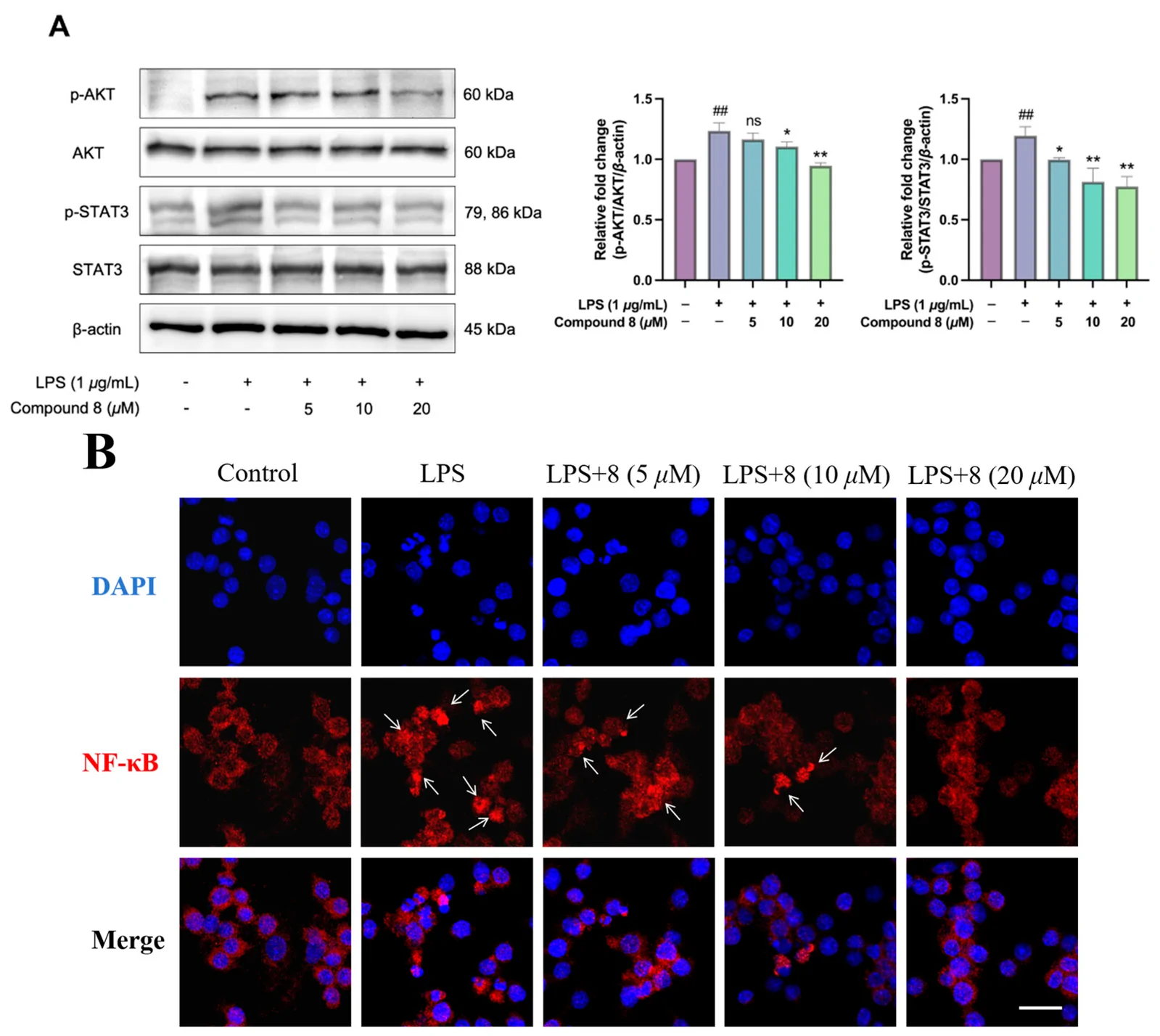
Pretreatment with compound **8** modulated AKT/STAT3 signaling pathway. RAW264.7 cells were treated with different concentrations (5, 10 and 20 μM) of compound **8** and LPS (1 μg/mL) for 24 h. (**A**) The effect of compound **8** on the Akt and STAT3 pathways in LPS induced RAW264.7 cells, with total Akt and total STAT3 as sample controls. (**B**) The NF-κB nuclear localizations of the treated cells were visualized through immunofluorescence analysis with an anti-NF-κB p65 (red) antibody. The nuclei were DAPI-stained (blue). Scale bar: 50 μm. Blue = nuclei, red = NF-κB, arrows = NF-κB nuclear translocation. Data are expressed as three independent experiments mean ± SD. ns: not significant; ## *p* < 0.01, compared with the control group. * *p* < 0.05, ** *p* < 0.01, compared with the LPS group.

Protein kinase B (Akt/PKB), a serine/threonine protein kinase, plays a crucial role in the activation of various signaling pathways, including STAT3. Previous studies have shown that Akt regulates transcriptional activity of STAT3 by inducing its phosphorylation [29]. To explore the effect of compound **8** on Akt signaling, we assessed the phosphorylation levels of Akt in LPS-treated cells. As shown in Figure 8A, pretreatment with compound **8** attenuated LPS-induced inflammatory responses and was accompanied by reduced phosphorylation of AKT and STAT3, suggesting a possible association between its anti-inflammatory effects and the AKT/STAT3 signaling pathway.

In addition, NF-κB is one of the most classical pathways regulating the initiation and progression of inflammation. Once activated, the NF-κB p65 subunit is released from the combination with inhibitory subunit IκB and subsequently translocated into the nucleus to engage in the transcription of multiple target genes, ultimately resulting in the release of proinflammatory factors. NF-κB expression correlates with elevated total cellular levels of STAT3 and supports expression and activation of STAT3 [30]. As shown in Figure 8B, confocal immunofluorescence analysis showed that pretreatment with compound **8** reduced the LPS-induced nuclear accumulation of NF-κB p65.

## 3. Materials and Methods

### 3.1. General Experimental Procedures

Infrared (IR) spectra were acquired using a Nicolet 6700 spectrophotometer (Thermo Fisher Scientific, Waltham, MA, USA). High-resolution electrospray ionization mass spectrometry (HR-ESI-MS) spectra were obtained on a Bruker Daltonics Apex-Ultra 7.0 T instrument (Bruker Corporation, Billerica, MA, USA). Nuclear magnetic resonance (NMR) spectra were measured using a Bruker AV-400 spectrometer (Bruker Switzerland AG, Fällanden, Switzerland). Optical rotations were recorded using a Modular Circular Polarimeter (Anton Paar GmbH, Graz, Austria, MCP 5100). Semi-preparative HPLC was performed on an LabTotal LC-20A (Shimadzu Corporation, Kyoto, Japan) series with a DAD detector using an Agilent Eclipse XDB-C18 (Agilent Technologies, Santa Clara, CA, USA) column (250 × 9.4 mm, 5 µm).

### 3.2. Fungal Material

*Chaetomium globosum* F0831 (GenBank accession number QB007695) was isolated from a marine sediment sample collected in the northern part of the South China Sea (GPS coordinates 115.1540° E, 20.1404° N) at a water depth of 500 m in July 2020. This fungal strain is currently preserved at the Key Laboratory of Tropical Medicinal Resource Chemistry, Ministry of Education, Hainan Normal University.

Fermentation, extraction and isolation. Two hundred Erlenmeyer flasks (1000 mL each), containing sterile solid medium composed of 75 g of rice and 120 mL of water with 3‰ sea salt, were inoculated with the *Chaetomium globosum* F0831 strain and incubated at 28 °C for 35 days. The rice-based medium underwent exhaustive extraction using EtOAc, followed by vacuum concentration to yield a dry EtOAc extract (230.0 g).

The EtOAc extract was subjected to silica gel column chromatography with a gradient elution of petroleum ether and ethyl acetate (100:0 to 0:100), yielding six fractions (Fr.A–Fr. F). Fr. C was further fractionated using reversed-phase column chromatography eluted with MeOH–H_2_O (30:70 to 100:0), affording four subfractions (Fr. C.1–Fr. C.4). Fr. C.2 was subsequently separated on a Sephadex LH-20 column (MeOH), giving three subfractions (Fr. C.2.1–Fr. C.2.3). Purification of Fr. C.2.1 by semi-preparative HPLC with CH_3_CN–H_2_O (36:64) yielded compounds **2** (5.2 mg) and **3** (10.3 mg). Similarly, Fr. C.2.3 was subjected to semi-preparative HPLC (CH_3_CN–H_2_O, 65:35) to afford compounds **8** (30.1 mg), **9** (50.0 mg), and **10** (15.0 mg). Fr. C.4 was chromatographed over a Sephadex LH-20 column eluted with MeOH, resulting in three subfractions (Fr. C.4.1–Fr. C.4.3). From Fr. C.4.2, compounds **1** (25.2 mg) and **4** (11.0 mg) were obtained via semi-preparative HPLC (CH_3_CN–H_2_O, 35:65). Fr. C.4.3 was purified by semi-preparative HPLC (CH_3_CN–H_2_O, 51:49) to yield compounds **11** (10.2 mg), **12** (11.7 mg), and **13** (15.3 mg). Fr. D was fractionated using reversed-phase column chromatography (MeOH–H_2_O, 20:80 to 100:0), providing five subfractions (Fr. D.1–Fr. D.5). Fr. D.3 was further separated on a Sephadex LH-20 column (MeOH), giving four subfractions (Fr. D.3.1–Fr. D.3.4). Final purification of Fr. D.3.3 by semi-preparative HPLC (CH_3_CN–H_2_O, 33:67) afforded compounds **5** (6.1 mg), **6** (15.6 mg), and **7** (12.0 mg).

X-Ray Crystallographic Data for Compound **1**. The crystals of compound **1** were obtained by slow evaporation from a MeOH solution. The crystallographic data for compound **1** have been deposited in the Cambridge Crystallographic Data Centre (CCDC) with the deposition number CCDC 2588332.

Crystal Data for C_12_H_16_O_4_ (*M* = 224.26 g/mol): orthorhombic, space group C222_1_, *a* = 7.0987(3) Å, *c* = 15.4116(6) Å, *V* = 15.4116(6) Å^3^, *Z* = 8, *T* = 293 K, μ(Cu Kα) = 0.803 mm^−1^, *Dcalc* = 1.2974 g/cm^3^, 6467 reflections measured (8.42° ≤ 2Θ ≤ 133.78°), 2037 unique (*R*_int_ = 0.0812, R_sigma_ = 0.0547), which were used in all calculations. The final *R*_1_ was 0.0606 (I > 2σ(I)), and *wR*_2_ was 0.1524 (all data).

(*S*)-1-(2,4-dihydroxy-3,5-dimethylphenyl)-3-hydroxybutan-1-one (**1**): colorless block-shaped crystals; mp 129.6–130.3 °C; [*α*${]}_{D}^{20}$ + 27.8 (*c* 0.1, MeOH); IR (KBr) *ν*_max_ 3443, 2833, 1599, 1360 cm^−1^; HR-ESI-MS *m*/*z* 247.0930 [M + Na]^+^ (calcd for C_12_H_16_NaO_4_^+^, 247.0941). For ^1^H and ^13^C NMR data, see Table 1.

5-[(8*S*)-2-hydroxypropyl]- 6-methylbenzene-1,3-diol (**2**): brown oily substance; [*α*${]}_{D}^{20}$ −13.5 (*c* 0.1, MeOH); IR (KBr) *ν*_max_ 3446, 1056, 1028, 1008 cm^−1^; HR-ESI-MS *m*/*z* 181.0865 [M-H]—(calcd for C_10_H_13_O_3_^−^, 181.0870). For ^1^H and ^13^C NMR data, see Table 1.

(*S*)-3,5,7-trimethyl-3,4-dihydrobenzo[c] [1,2] dioxine-6,8-diol (**3**): brown oily substance; [*α*${]}_{D}^{20}$ −10.5 (*c* 0.1, MeOH); IR (KBr) *ν*_max_ 3420, 1652, 1635, 1120, 823 cm^−1^; HR-ESI-MS *m*/*z* 209.0819 [M-H]—(calcd for C_11_H_13_O_4_^−^, 209.0819). For ^1^H and ^13^C NMR data, see Table 1.

7-O-acetylarmochaetoglobin Q (**5**): brown oily substance; [*α*${]}_{D}^{20}$ −135.3 (*c* 0.1, MeOH); IR (KBr) *ν*_max_ 3445, 2832, 1609, 1364, 779 cm^−1^; HR-ESI-MS *m*/*z* 587.2770 [M-H]—(calcd for C_34_H_39_N_2_O_7_, 587.2762). For ^1^H and ^13^C NMR data, see Table 2.

### 3.3. Computational Section

Conformational analysis of all compounds was performed using Spartan 14 software with the MMFF94S force field. The conformers were then imported into the Gaussian 16 package. Geometry optimization was carried out at the B3LYP/6-31+G(d) level. The optimized conformers were subjected to electronic circular dichroism (ECD) calculations using time-dependent density functional theory (TDDFT) at the B3LYP/6-311G* level in methanol, with the polarizable continuum model (PCM) employed to account for solvation effects. The calculated ECD curves were generated using SpecDis 1.71. The calculated spectra were Boltzmann-weighted according to the relative populations of the low-energy conformers in MeOH.

### 3.4. Cell Culture and Treatment

The RAW 264.7 cells were purchased from the Chinese Academy of Sciences Cell Bank (Shanghai, China).RAW 264.7 cells were maintained in Dulbecco’s Modified Eagle Medium (DMEM) (C11995500BT, Gibco, Waltham, MA, USA), supplemented with 10% (*v*/*v*) fetal bovine serum (FBS) (10099141C, Gibco, Waltham, MA, USA) and 1% penicillin–streptomycin (PS) (15140122, Gibco, Waltham, MA, USA). Cells were incubated in a humidified incubator with 5% CO_2_ and 95% air at 37 °C. To evaluate the anti-inflammatory effects of compound **8**, RAW 264.7 cells were seeded at a density of 2 × 10^4^ cells per well in a 96-well plate. The cells were treated with 1 μg/mL lipopolysaccharide (LPS) in the presence or absence of compound **8** (20 μM) for 12 h. Inflammatory mediator production, including nitric oxide (NO), and pro-inflammatory cytokines (TNF-*α*, IL-6, IL-1*β*), was measured to evaluate the inhibitory effects of compound **8** on LPS-stimulated inflammatory responses.

### 3.5. Determination of NO Production

Nitric oxide (NO) production was evaluated by measuring nitrite (NO_2_^−^) levels in the culture supernatants using the Griess Reagent method with a commercial Nitric Oxide Assay Kit (Beyotime, S0021, Shanghai, China), according to the manufacturer’s instructions. Briefly, culture supernatants were collected after treatment and centrifuged when necessary to remove cellular debris. A NaNO_2_ standard curve was prepared using concentrations of 0, 1, 2, 5, 10, 20, 40, 60, and 100 μM in the same culture medium used for the samples. Subsequently, 50 μL of each standard or sample was mixed sequentially with 50 μL of Griess Reagent I and 50 μL of Griess Reagent II in a 96-well plate. After incubation at room temperature, absorbance was measured at 540 nm. Nitrite concentrations were calculated from the NaNO_2_ standard curve (R^2^ > 0.99).

Cell viability was evaluated in parallel using the CCK-8 assay to assess potential cytotoxic interference. NO production was normalized to the LPS-treated group, which was defined as 100%. Relative NO production was calculated as: relative NO production (%) = (NO_2_^−^ concentration of sample/NO_2_^−^ concentration of LPS group) × 100. The NO inhibition rate was calculated as: inhibition rate (%) = [(NO_2_^−^ concentration of LPS group − NO_2_^−^ concentration of sample)/(NO_2_^−^ concentration of LPS group − NO_2_^−^ concentration of control group)] × 100. All experiments were performed independently at least three times, and the results are expressed as the mean ± SD.

### 3.6. ELISA Assay

To evaluate cytokine concentrations in the cell culture supernatant, RAW264.7 cells were cultured under the indicated experimental conditions. The concentration of TNF-*α* (RayBiotech, Peachtree Corners, GA, USA, #ELM-TNF*α*), IL-1*β* (RayBiotech, Peachtree Corners, GA, USA, #ELM-IL-1b), and IL-6 (RayBiotech, Peachtree Corners, GA, USA, #ELM-IL6) in cell culture supernatant was detected using ELISA kits according to manufacturers’ instructions. Briefly, the assay was based on a double-antibody sandwich method, and the absorbance of samples was measured at 450 nm using a microplate reader.

### 3.7. Intracellular ROS Detection by Fluorescence Microscopy

RAW264.7 cells were pretreated with compound 8 (5, 10, or 20 μM) for 1 h and then stimulated with LPS (1 μg/mL) for 24 h. Intracellular ROS were detected using the DCFH-DA fluorescent probe. Following treatment, cells were incubated with DCFH-DA (diluted 1:1000) at 37 °C for 30 min and subsequently washed with serum-free culture medium. Fluorescence images were acquired using an Olympus FV3000 confocal laser scanning microscope (Olympus, Tokyo, Japan) under identical imaging settings for all groups. Fluorescence intensity was quantified using ImageJ 1.53c. Background fluorescence was subtracted, and the mean fluorescence intensity within the selected regions of interest (ROIs) was calculated and normalized to that of the control group.

### 3.8. Western Blotting Analysis

Cells were lysed in RIPA buffer (Beyotime, Shanghai, China) with added protease inhibitors for 30 min on ice. The resulting homogenates were then centrifuged at 12,000 rpm for 10 min at 4 °C. Total protein concentrations in the supernatants were measured using the BCA assay. Protein samples, adjusted to equal concentrations, were separated on SDS-PAGE gels and subsequently transferred to PVDF membranes (Millipore, Burlington, MA, USA). After a 2-h sealing treatment with 5% BSA, the membrane was further incubated at 4 °C overnight to allow it to bind to the specific antibodies related to the target protein. After washing, the membranes were incubated with the corresponding secondary antibodies for 2 h at room temperature (RT). The immune complexes were detected with an ECL reagent (Millipore, Burlington, MA, USA). Following incubation with horseradish peroxidase (HRP)-conjugated secondary antibodies at room temperature. Immunoblots were analyzed using analytikjena (Analytik Jena, Burlington, MA, USA).

### 3.9. Immunofluorescence Staining

RAW264.7 cells were seeded on coverslips and cultured overnight. After the indicated treatments, the cells were fixed with 4% paraformaldehyde for 15 min, permeabilized with 0.1% Triton X-100 for 10 min, and blocked with 5% bovine serum albumin (BSA) for 1 h at room temperature. The cells were then incubated with an anti-NF-κB p65 primary antibody overnight at 4 °C, followed by incubation with an appropriate fluorescence-conjugated secondary antibody for 1 h at room temperature in the dark. Nuclei were counterstained with DAPI. Fluorescence images were acquired using an Olympus FV3000 confocal laser scanning microscope (Olympus, Tokyo, Japan) under identical imaging settings for all groups.

### 3.10. Statistical Analysis

Data were analyzed using GraphPad Prism 10.4. The figures and/or legends contained statistical tests, n values, replicate experiments, and *p* values. A two-tailed Student’s t-test was used to assess significance between the two groups, while one-way ANOVA with Tukey’s post hoc test was employed to evaluate significance among multiple groups. Statistical significance was defined as a *p* value < 0.05. Error bars indicate mean ± SD. A difference at * *p* < 0.05 was considered significant, and that at ** *p* < 0.01 was considered highly significant. Ns: not significant.

## 4. Conclusions

In our continuing search for bioactive secondary metabolites from marine-derived fungi, a chemical investigation of *Chaetomium globosum* obtained from marine sediments led to the isolation of four undescribed compounds (**1**–**3** and **5**), representing diverse structural classes including two resorcinol derivatives, a rare benzo[c][1,2]dioxine endoperoxide, and a cytochalasan alkaloid, together with nine known congeners. Bioassay results demonstrated that compound **8** was found to attenuate the inflammatory response, potentially by inhibiting the AKT/STAT3 signaling pathway, thereby suppressing the expression of inflammatory mediators (iNOS and COX-2) and pro-inflammatory cytokines (IL-6, TNF-*α*, and IL-1*β*). SAR analysis of the chaetomugilin class indicates that the C-1 lactone ring and the C-8 hydroxy group are favorable for anti-inflammatory activity. Chaetomugilins E and F, which retain both features, inhibited NO production with IC_50_ values of 5.8 and 1.9 μM, respectively [31]. Similarly, chaetomugilin I and 11-epi-chaetomugilin I suppressed TNF-α-induced NF-κB activity (IC_50_ = 0.9 μM) and NO release (IC_50_ = 0.3 and 0.8 μM, respectively) [32]. In contrast, chaephilones C and D, lacking these features, showed only moderate NO inhibition (IC_50_ = 15.12 ± 1.48 and 20.65 ± 0.95 μM) [33]. In the present study, compound **8** (IC_50_ = 10.99 ± 0.17 μM) also exhibited greater activity than compound **9** (IC_50_ = 28.56 ± 0.39 μM), consistent with the favorable roles of the lactone ring and C-8 hydroxy group. Among the compounds isolated in this work, compound **8** was the most potent NO inhibitor, and its C-1 lactone ring and C-8 hydroxy group may provide a structural basis for further optimization of azaphilone-based anti-inflammatory leads. These findings also expand the chemical diversity of *C. globosum*.

## Figures and Tables

**Table 1:** ^1^H (*J* in Hz, *δ*_H_ in ppm) NMR and ^13^C (*δ*_C_ in ppm) NMR data of compounds **1** in CD_3_OD, **2** and **3** in DMSO-*d*_6_.

	1		2		3	
Position	*δ*_H_ (*J* in Hz)	*δ*_C_ (ppm)	*δ*_H_ (*J* in Hz)	*δ*_C_ (ppm)	*δ*_H_ (*J* in Hz)	*δ*_C_ (ppm)
1		111.9		155.8		146.1
2		162.6	6.13 (s, 1H)	100.3		112.2
3		113.8		154.9		136.3
4		162.2	6.02 (s, 1H)	108.3		139.9
5		117.2		139.3		123.2
6	7.45 (s, 1H)	130.7		112.7		111.5
7		204.6	2.34 (m, 1H), 2.65 (m, 1H)	43.5	2.64 (m, 1H), 3.15 (m, 1H)	36.9
8	2.92 (dd, 15.6, 5.0, 1H) 3.11 (dd, 15.6, 7.5, 1H)	47.6	3.73 (m, 1H)	66.7	4.79 (m, 1H)	78.6
9	4.33 (m, 1H)	65.7	1.00 (d, 6.1, 3H)	23.1	1.37 (d, 6.3, 3H)	21.7
10	1.25 (d, 6.3, 3H)	23.4				
11	2.05 (s, 3H)	8.0				
12	2.16 (s, 3H)	16.4				
2-CH_3_					1.95 (s, 3H)	9.7
6-CH_3_			1.94 (s, 3H)	11.0	1.96 (s, 3H)	12.9
1-OH					7.40 (s, 1H)	
3-OH					8.23 (s, 1H)	

**Table 2:** ^1^H NMR and ^13^C NMR data of compound **5** (*J* in Hz, *δ* in ppm) in CD_3_OD.

	5	
Position	*δ*_H_ (*J* in Hz)	*δ*_C_ (ppm)
1		174.9
2		
3	3.77 (q, 4.9, 1H)	55.5
4	3.11 (t, 4.8, 1H)	43.3
5	2.02 (m, 1H)	41.7
6		73.6
7	4.84 (d, 11.4, 1H)	75.0
8	2.56 (t, 11.4, 1H)	47.1
9		64.0
10	2.76 (dd, 14.6, 6.0, 1H) 2.89 (dd, 14.6, 6.0, 1H)	33.2
11	1.12 (d, 7.2, 3H)	13.7
12	1.17 (s, 3H)	24.7
13	5.80 (dd, 14.6, 11.1, 1H)	128.8
14	5.16 (ddd, 14.6, 11.1, 3.6, 1H)	135.4
15	1.92 (m, 1H) 2.23 (m, 1H)	43.1
16	2.49 (m, 1H)	33.3
17	5.58 (d, 9.3, 1H)	141.6
18		133.9
19	5.13 (s, 1H)	82.6
20		201.8
21	6.38 (d, 16.7, 1H)	133.4
22	7.84 (d, 16.7, 1H)	137.7
23		198.7
24 (16-Me)	0.98 (d, 6.8, 1H)	21.2
25 (18-Me)	1.35 (s, 3H)	10.9
7-OCOCH_3_		172.4
7-OCOCH_3_	1.98 (s, 3H)	20.8
1′-NH		
1′a		128.8
2′	7.03 (s, 1H)	122.4
3′a		138.0
3′		110.6
4′	7.48 (d, 7.8, 1H)	119.3
5′	7.07 (m, 1H)	125.0
6′	6.99 (m, 1H)	119.9
7′	7.30 (d, 8.0, 1H)	112.4

## Data Availability

The raw data supporting the conclusions of this article will be made available by the authors on request.

## Supplementary Materials

The following supporting information can be downloaded at https://www.mdpi.com/article/10.3390/md24100355/s1. Figure S1−S8: ^1^H and ^13^C NMR, DEPT, HSQC, HMBC, ^1^H−^1^H COSY, IR, and (−)-HR-ESI-MS spectra of compound **1**; Figure S9−S16: 1D and 2D NMR, IR, and HR-ESI-MS spectra of compound **2**; Figure S17−S23: 1D and 2D NMR, IR, and (-)-HR-ESI-MS spectra of compound **3**; Figure S24−S31: 1D and 2D NMR, NOESY, IR, and (+)-HR-ESI-MS spectra of compound **5**; Figure S32−S33: Original, uncropped Western blot images corresponding to Figure 6D and Figure 8A; Table S1−S6: Crystallographic data and structure refinement, fractional atomic coordinates, bond lengths/angles, and hydrogen atom coordinates for compound **1**.
