# Supplementary material for: Secondary Metabolites from a Marine-Derived Fungus Chaetomium globosum F0831 and Their Anti-Inflammatory Effects
Source: Mar Drugs. 2026 Oct 9;24(10):355. doi: 10.3390/md24100355 (PMC13706603; doi:10.3390/md24100355)
Supplement: Supplementary file 1 [file marinedrugs-24-00355-s001.zip › marinedrugs-4600845-supplementary.pdf]

Secondary Metabolites from a Marine-Derived Fungus *Chaetomium globosum* F0831 and Their Anti-Inflammatory Effects

TuXiang Mo[1,2,3], Xuan Zhang[1,2,3], ChenYang Yi[1,2,3], Hua Wu[1,2,3], XinRui Xia[1,2,3], XinJie Ge[1,2,3], Hong Xiao[1,2,3], WanShan Li and GuangYing Chen[1,2,3],*

1 Key Laboratory of Tropical Medicinal Resource Chemistry of Ministry of Education, Haikou, Hainan 571158, People's Republic of China
2 Key Laboratory of Tropical Medicinal Plant Chemistry of Hainan Province, School of Chemistry and Chemical Engineering, Hainan Normal University, Haikou, Hainan 571158, People's Republic of China
3 Key Laboratory of Hainan Provincial International Joint Research Center for Comprehensive Utilization and Efficient Conversion of Tropical Medicinal Resources, Haikou, Hainan 571158, People's Republic of China
* Correspondence: mimowanshan@163.com (W.-S.L); chgying123@163.com (G.-Y.C.)

Contents of Supporting Information

Contents

Table S1 Crystal data and structure refinement for 1 3
Table S2 Fractional Atomic Coordinates (×104) and Equivalent Isotropic Displacement Parameters (Å2×103) for 1_auto. Ueq is defined as 1/3 of of the trace of the orthogonalised UIJ tensor......3
Table S3 Anisotropic Displacement Parameters (Å2×103) for 1_auto. The Anisotropic displacement factor exponent takes the form: -2π2[h2a*2U11+2hka*b*U12+…]. 4
Table S4 Bond Lengths for 1......4
Table S5 Bond Angles for 1 5
Table S6 Hydrogen Atom Coordinates (Å×104) and Isotropic Displacement Parameters (Å2×103) for 1_auto......5
Figure S1 ^{1}H NMR spectrum of compound 1 in CD_3OD. 6
Figure S2 ^{13}C NMR spectrum of compound 1 in CD_3OD. 7
Figure S3 DEPT135° NMR spectra of compound 1 in CD_3OD. 7
Figure S4 HSQC spectra of compound 1 in CD_3OD. 8
Figure S5 HMBC spectra of compound 1 in CD_3OD. 9
Figure S6 ^{1}H−^{1}H COSY spectra of compound 1 in CD_3OD......9
Figure S8 (-)-HR-ESI-MS spectrum of compound 1. 10
Figure S9 ^{1}H NMR spectrum of compound 2 in DMSO-d_6......11
Figure S10 ^{13}C NMR spectrum of compound 2 in DMSO-d_6......11
Figure S11 DEPT135° NMR spectra of compound 2 in DMSO-d_6......12
Figure S12 HSQC spectra of compound 2 in DMSO-d_6......12
Figure S13 HMBC spectra of compound 2 in DMSO-d_6......13
Figure S14 ^{1}H−^{1}H COSY spectra of compound 2 in DMSO-d_6. 13
Figure S15 IR data of 2 in KBr......13
Figure S16 (-)-HR-ESI-MS spectrum of compound 2. 14
Figure S17 ^{1}H NMR spectrum of compound 3 in DMSO-d_6......14
Figure S18 ^{13}C NMR spectrum of compound 3 in DMSO-d_6......15
Figure S19 HSQC spectra of compound 3 in DMSO-d_6......15
Figure S20 HMBC spectra of compound 3 in DMSO-d_6......16
Figure S21 ^{1}H−^{1}H COSY spectra of compound 3 in DMSO-d_6. 17
Figure S22 IR data of 3 in KBr......17
Figure S23 (-)-HR-ESI-MS spectrum of compound 3. 17
Figure S24 ^{1}H NMR spectrum of compound 5 in CD_3OD. 18
Figure S25 ^{13}C NMR spectrum of compound 5 in CD_3OD. 18
Figure S26 HSQC spectra of compound 5 in CD_3OD. 19
Figure S27 HMBC spectra of compound 5 in CD_3OD. 19
Figure S28 ^{1}H−^{1}H COSY spectra of compound 5 in CD_3OD......20
Figure S29 NOESY spectra of compound 5 in CD_3OD......21
Figure S30 IR data of 5 in KBr......21
Figure S31 (+)-HR-ESI-MS spectrum of compound 5. 22
Figure S32 Original, uncropped Western blot images corresponding to Figure 6 D. 22
Figure S33 Original, uncropped Western blot images corresponding to Figure 8 A......22

Table S1 Crystal data and structure refinement for 1

**Identification code**	**1**
Empirical formula	$C_{12}H_{16}O_4$
Formula weight	224.26
Temperature/K	293
Crystal system	orthorhombic
Space group	$C222_1$
a/Å	7.0987(3)
b/Å	20.9869(7)
c/Å	15.4116(6)
α/°	90
β/°	90
γ/°	90
Volume/$Å^3$	2296.02(15)
Z	8
$\rho_{calc}g/cm^3$	1.2974
μ/mm^{-1}	0.803
F(000)	963.4
Crystal size/mm^3	0.14 × 0.13 × 0.12
Radiation	Cu Kα (λ = 1.54184)
2Θ range for data collection/°	8.42 to 133.78
Index ranges	$-4 \leq h \leq 8$, $-24 \leq k \leq 24$, $-16 \leq l \leq 18$
Reflections collected	6467
Independent reflections	2037 [R_{int} = 0.0812, R_{sigma} = 0.0547]
Data/restraints/parameters	2037/0/150
Goodness-of-fit on F^2	0.996
Final R indexes [I>=2σ (I)]	R_1 = 0.0606, wR_2 = 0.1388
Final R indexes [all data]	R_1 = 0.0680, wR_2 = 0.1524
Largest diff. peak/hole / e $Å^{-3}$	0.36/-0.50
Flack parameter	0.0(4)

Table S2 Fractional Atomic Coordinates (×104) and Equivalent Isotropic Displacement Parameters (Å2× 103) for 1_auto. Ueq is defined as 1/3 of of the trace of the orthogonalised UIJ tensor.

**Atom**	***x***	***y***	***z***	**U(eq)**
C4	-2537(4)	-5073.7(9)	-7393.5(14)	45.7(5)
C5	-2525(4)	-6242(1)	-7727.7(13)	46.9(5)

C6	-2514(4)	-5739.8(9)	-7126.2(12)	44.4(4)
C7	-2515(4)	-6870.2(9)	-7489.1(15)	50.4(5)
C8	-2506(5)	-4897.5(9)	-8342.1(14)	50.4(5)
C9	-2443(5)	-6533.9(10)	-5964.2(15)	52.9(5)
C10	-2470(4)	-5899.5(9)	-6237.7(13)	48.3(5)
C11	-2496(4)	-7006.0(9)	-6589.7(14)	51.2(5)
C12	-2390(6)	-6700.9(13)	-5017.7(17)	72.9(7)
C13	-2903(3)	-4199.6(11)	-8543.0(17)	55.0(6)
C14	-2508(6)	-7394.9(11)	-8150.7(17)	66.6(6)
C16	-2731(6)	-4074.1(13)	-9505.6(17)	74.9(9)
O1	-2566(3)	-4639.1(7)	-6846.4(11)	58.5(4)
O2	-2474(4)	-7609.4(7)	-6278.8(12)	70.7(5)
O3	-2456(5)	-5453.5(7)	-5609.3(10)	68.9(5)
O17	-1622(3)	-3783.4(8)	-8100.8(14)	62.6(5)

Table S3 Anisotropic Displacement Parameters (Å2×103) for 1_auto. The Anisotropic displacement factor exponent takes the form: -2π2[h2a*2U11+2hka*b*U12+⋯].

Atom	U_{11}	U_{22}	U_{33}	U_{12}	U_{13}	U_{23}
C4	55.7(9)	34.3(10)	47.2(10)	-0.9(11)	-0.4(13)	-3.1(8)
C5	61.8(11)	37.5(10)	41.2(9)	0.9(13)	-0.1(13)	-3.3(8)
C6	58.0(9)	30.0(9)	45(1)	-1.5(11)	-1.3(13)	-2.8(8)
C7	66.7(11)	32.7(10)	51.9(12)	-3.2(13)	0.2(16)	-8.2(9)
C8	71.5(11)	30.8(10)	48.7(11)	0.3(12)	1.5(14)	0.1(8)
C9	77.6(12)	35.1(11)	46.1(11)	-3.3(15)	-1.9(14)	0.3(8)
C10	68.5(11)	34.1(10)	42.3(10)	-3.5(12)	-1.3(13)	-6.1(8)
C11	71.1(11)	31.7(10)	50.8(11)	-0.2(12)	-3.1(14)	-0.3(8)
C12	121(2)	49.0(13)	48.1(13)	-4.3(19)	-8(2)	3.3(10)
C13	73.4(15)	35.6(11)	56.0(12)	-0.7(9)	-6.0(11)	1.3(10)
C14	103.1(17)	40.6(12)	56.1(13)	-1.5(15)	1(2)	-14.5(10)
C16	121(3)	47.3(14)	56.4(14)	-5.0(18)	-11.4(18)	8.8(11)
O1	92.1(11)	32.4(7)	51.1(8)	1.8(9)	-0.2(12)	-6.4(6)
O2	120.2(14)	30.8(8)	61(1)	-4.1(11)	-10.2(15)	1.9(7)
O3	127.9(15)	36.5(9)	42.2(8)	-2.2(13)	-1.7(13)	-9.8(6)
O17	91.1(11)	34.4(8)	62.3(11)	-7.6(8)	-14.3(10)	3.4(8)

Table S4 Bond Lengths for 1.

Atom	Atom	Length/Å	Atom	Atom	Length/Å
C4	C6	1.457(3)	C8	C13	1.523(3)

C4	C8	1.508(3)	C9	C10	1.397(3)
C4	O1	1.242(3)	C9	C11	1.383(3)
C5	C6	1.404(3)	C9	C12	1.501(3)
C5	C7	1.369(3)	C10	O3	1.347(2)
C6	C10	1.410(3)	C11	O2	1.354(3)
C7	C11	1.415(3)	C13	C16	1.512(4)
C7	C14	1.501(3)	C13	O17	1.433(3)

Table S5 Bond Angles for 1

Atom	Atom	Atom	Angle/°	Atom	Atom	Atom	Angle/°
C8	C4	C6	120.60(17)	C12	C9	C10	121.1(2)
O1	C4	C6	120.84(19)	C12	C9	C11	120.7(2)
O1	C4	C8	118.56(18)	C9	C10	C6	121.33(18)
C7	C5	C6	123.08(19)	O3	C10	C6	122.23(18)
C5	C6	C4	122.24(18)	O3	C10	C9	116.45(18)
C10	C6	C4	120.18(18)	C9	C11	C7	122.60(19)
C10	C6	C5	117.58(18)	O2	C11	C7	122.35(19)
C11	C7	C5	117.20(19)	O2	C11	C9	115.02(19)
C14	C7	C5	121.6(2)	C16	C13	C8	110.6(2)
C14	C7	C11	121.2(2)	O17	C13	C8	111.8(2)
C13	C8	C4	115.47(19)	O17	C13	C16	108.0(2)
C11	C9	C10	118.18(19)				

Table S6 Hydrogen Atom Coordinates (Å×104) and Isotropic Displacement Parameters (Å2×103) for 1_auto.

Atom	*x*	*y*	*z*	U(eq)
H5	-2539(4)	-6143.2(10)	-8316.0(13)	56.2(6)
H8a	-1279(5)	-5005.8(9)	-8576.0(14)	60.4(6)
H8b	-3432(5)	-5156.1(9)	-8642.7(14)	60.4(6)
H12a	-1193(15)	-6888(11)	-4880(4)	109.3(11)
H12b	-2570(40)	-6322(2)	-4678.0(17)	109.3(11)
H12c	-3380(20)	-6999(10)	-4890(4)	109.3(11)
H13	-4190(3)	-4098.5(11)	-8358.8(17)	66.0(7)
H14a	-2550(40)	-7214.0(12)	-8722.5(18)	99.9(10)
H14b	-1380(20)	-7644(9)	-8087(13)	99.9(10)
H14c	-3590(20)	-7662(8)	-8066(12)	99.9(10)
H16a	-1498(15)	-4196(13)	-9700(4)	112.4(14)
H16b	-3660(30)	-4318(11)	-9811(2)	112.4(14)
H16c	-2920(40)	-3629(3)	-9617(3)	112.4(14)

H2	-2780(60)	-7857(2)	-6664(9)	106.0(8)
H3	-2500(70)	-5098.3(10)	-5829.9(15)	103.3(8)
H17	-1640(40)	-3862(13)	-7580(4)	94.0(8)

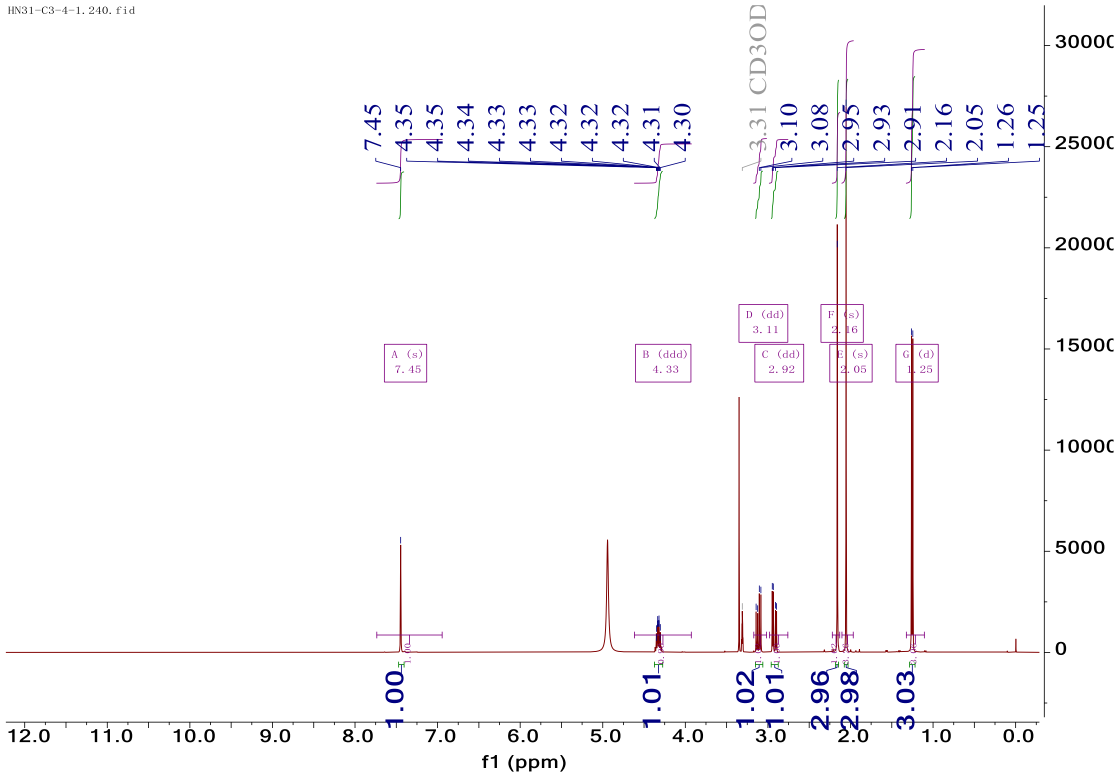

Figure S1 ^{1}H NMR spectrum of compound **1** in CD_3OD.

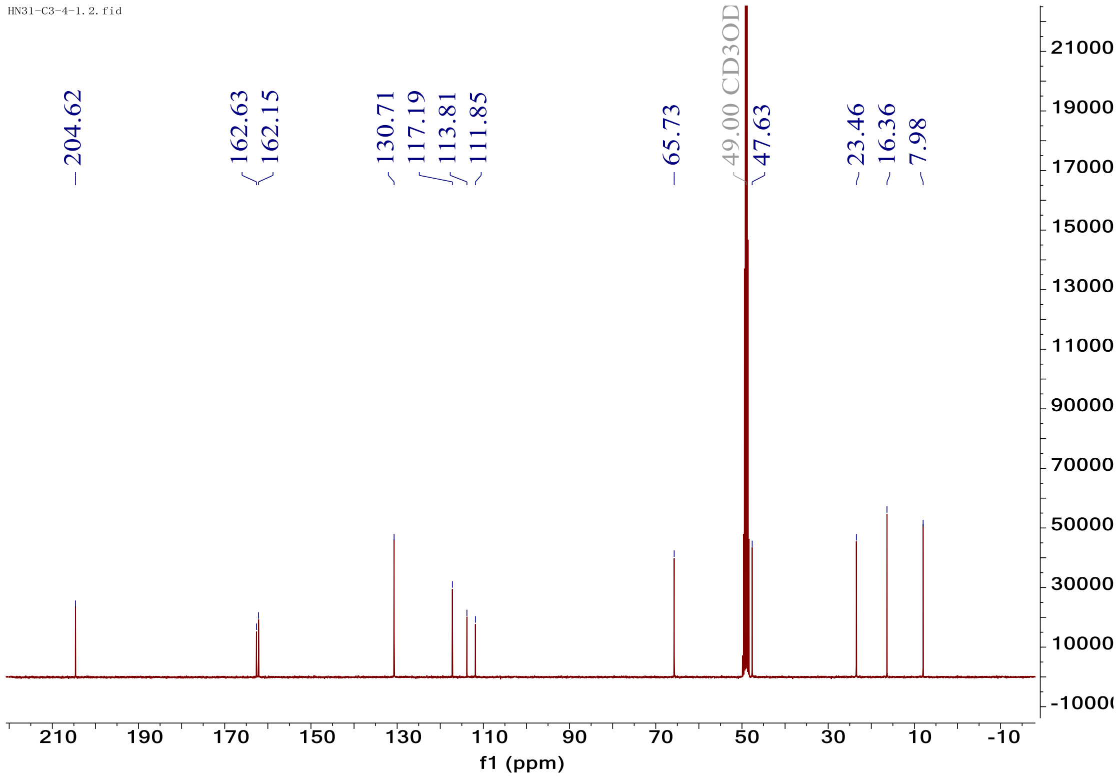

Figure S2 ^{13}C NMR spectrum of compound **1** in CD_3OD.

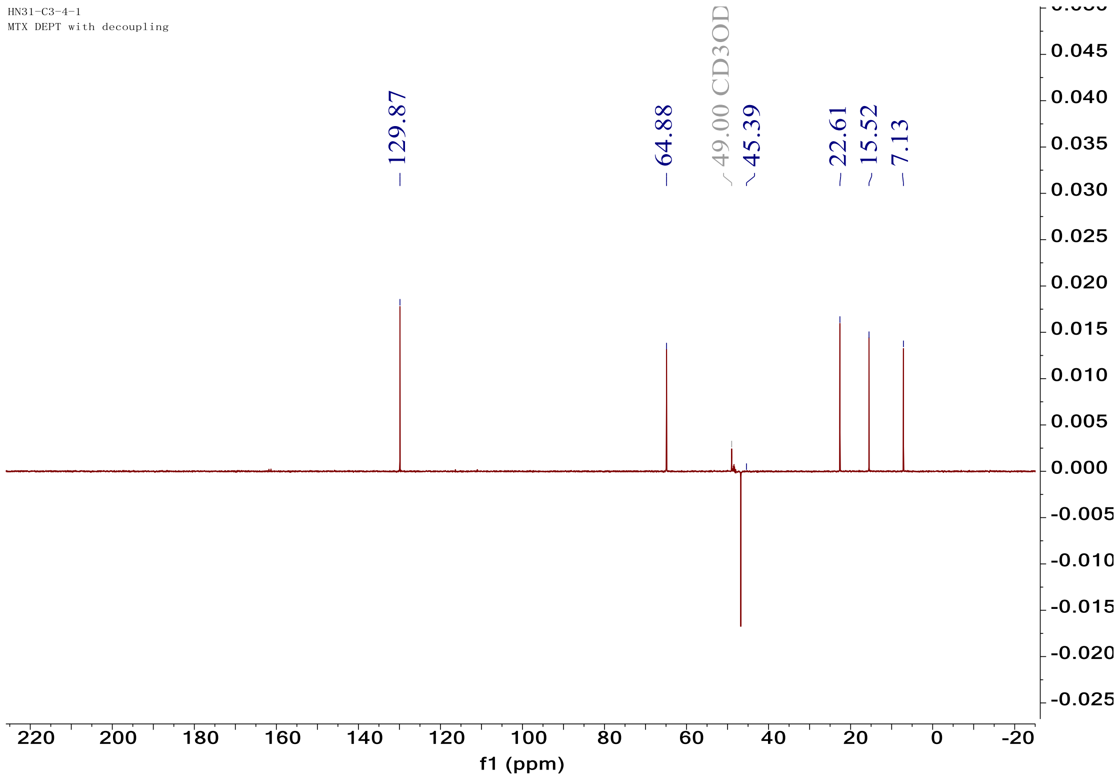

Figure S3 DEPT135° NMR spectra of compound **1** in CD_3OD.

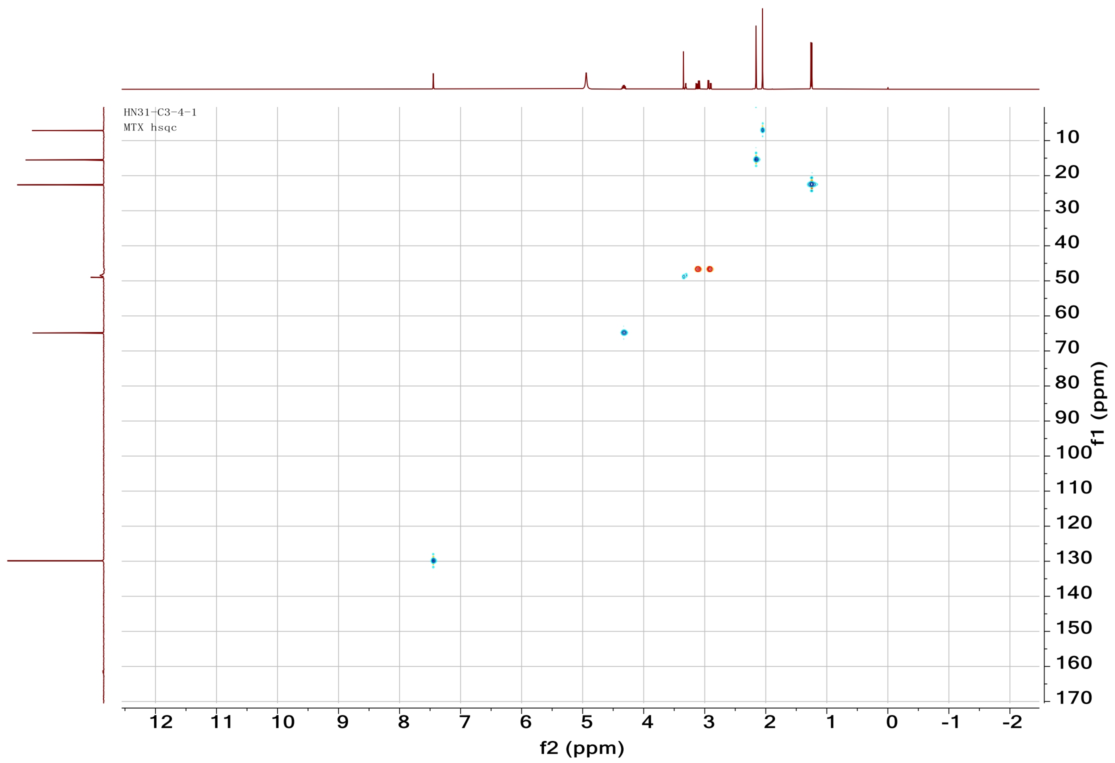

Figure S4 HSQC spectra of compound **1** in CD_3OD.

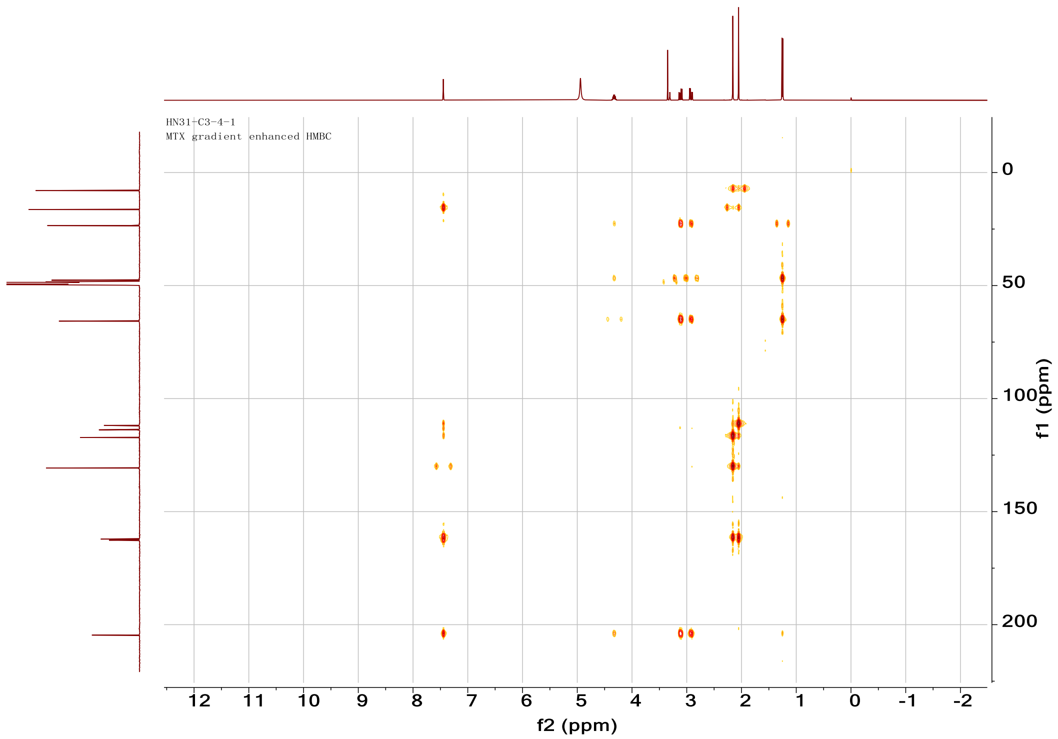

Figure S5 HMBC spectra of compound **1** in CD_3OD.

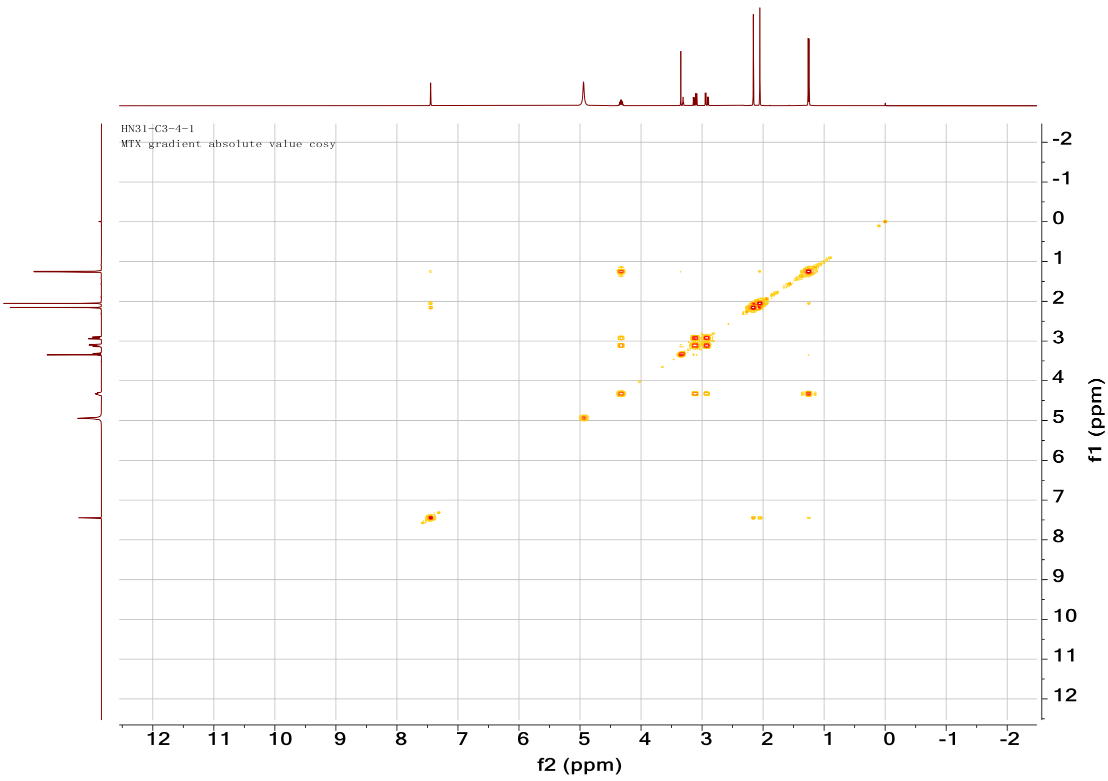

Figure S6 ^{1}H–^{1}H COSY spectra of compound **1** in CD_3OD.

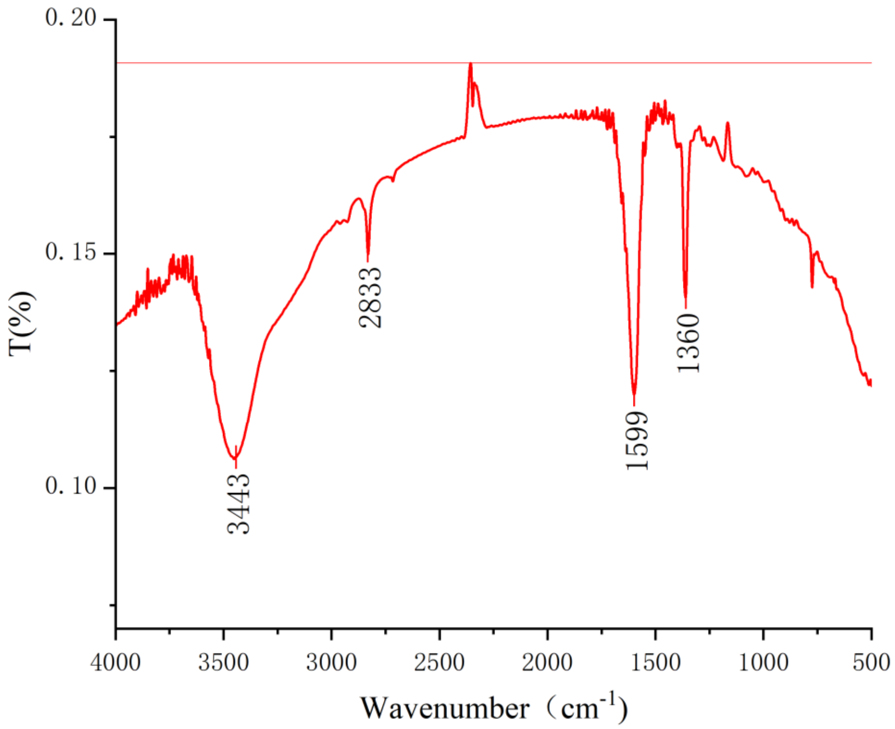

Figure S7 IR data of **1** in KBr

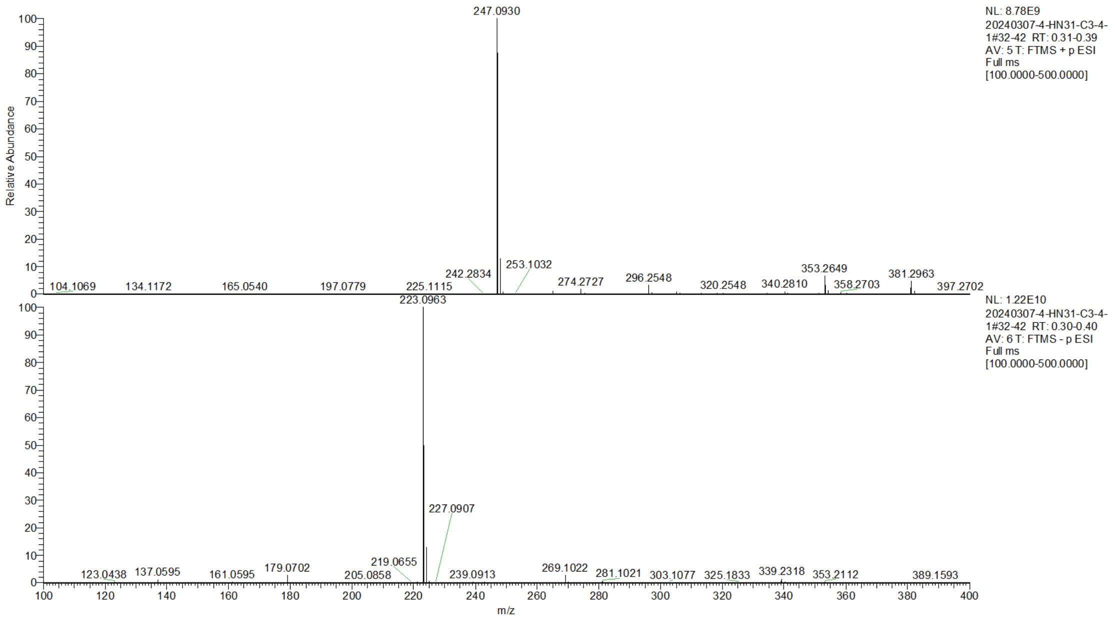

Figure S8 (-)-HR-ESI-MS spectrum of compound **1**.

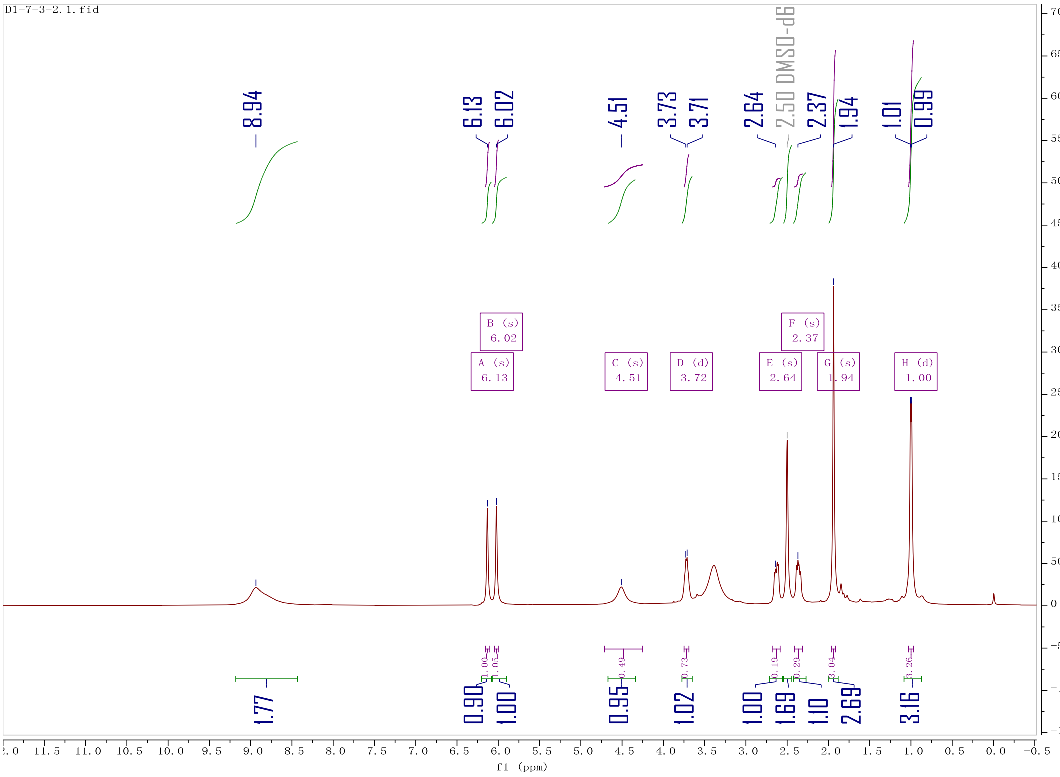

Figure S9 ^{1}H NMR spectrum of compound **2** in DMSO-d_6.

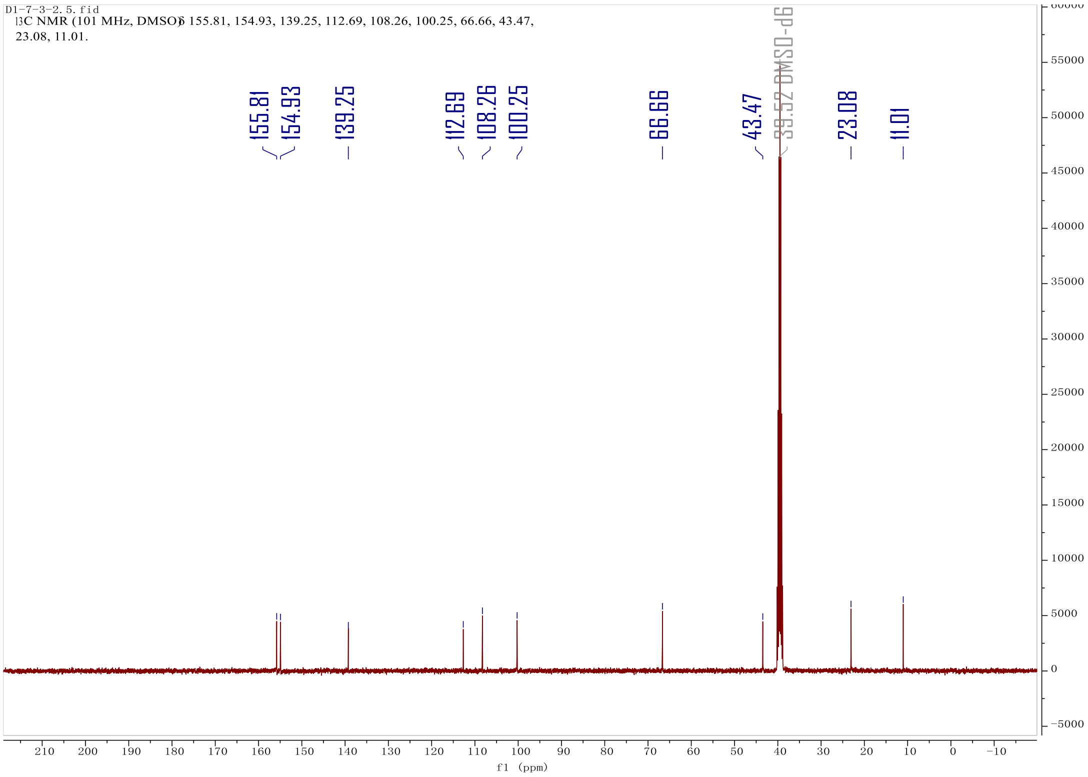

Figure S10 ^{13}C NMR spectrum of compound **2** in DMSO-d_6.

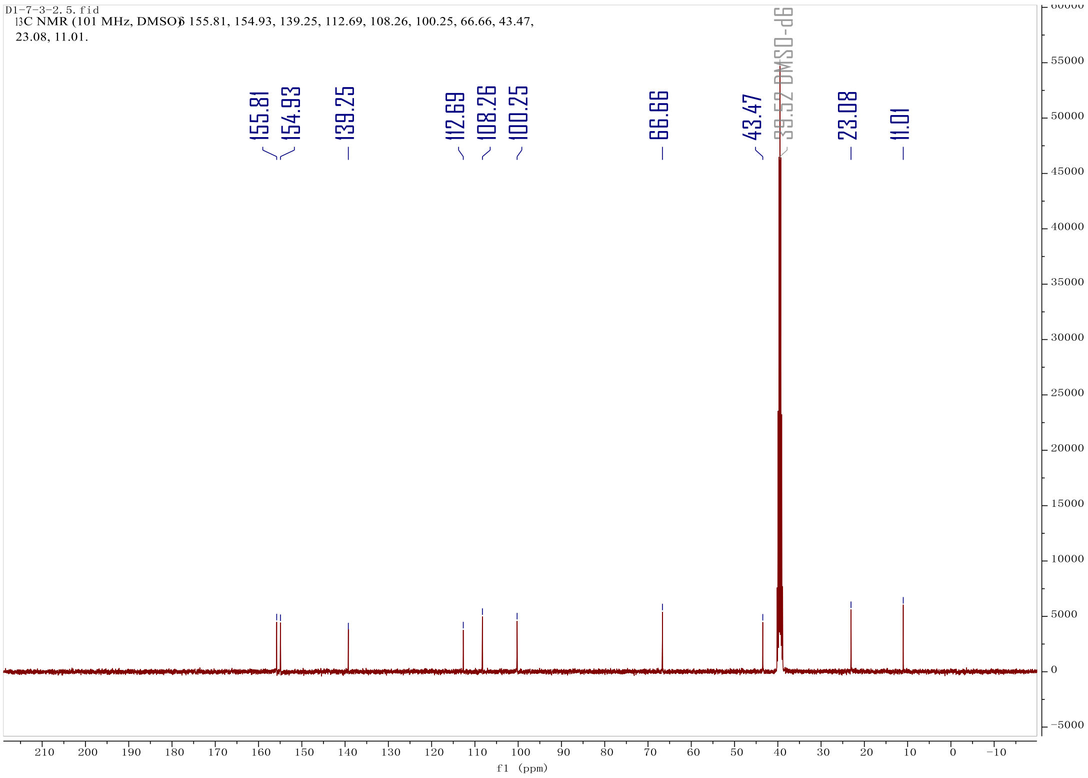

Figure S11 DEPT135° NMR spectra of compound **2** in DMSO-d_6.

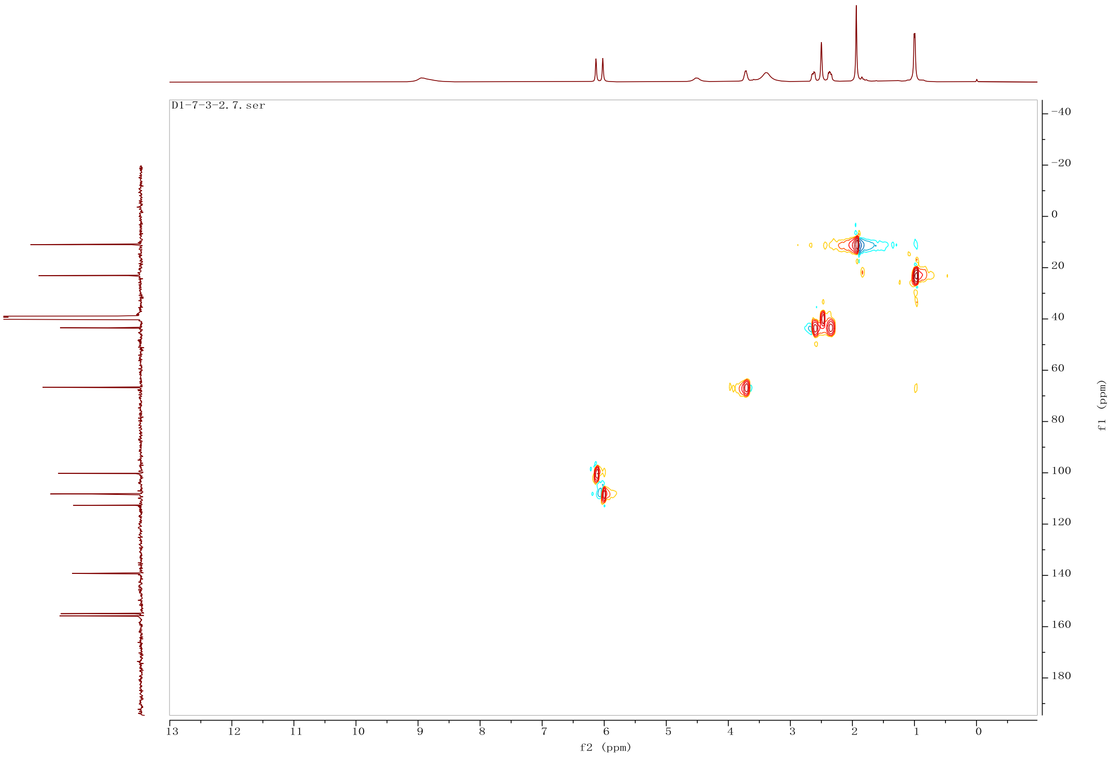

Figure S12 HSQC spectra of compound **2** in DMSO-d_6.

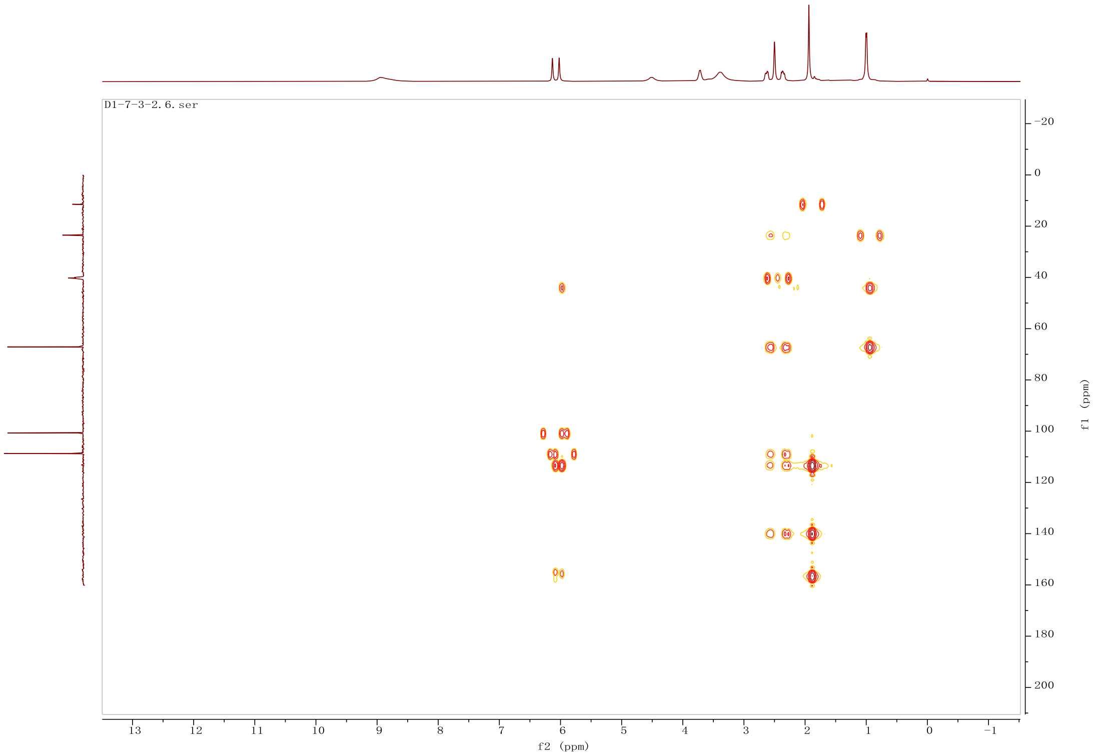

Figure S13 HMBC spectra of compound **2** in DMSO-d_6.

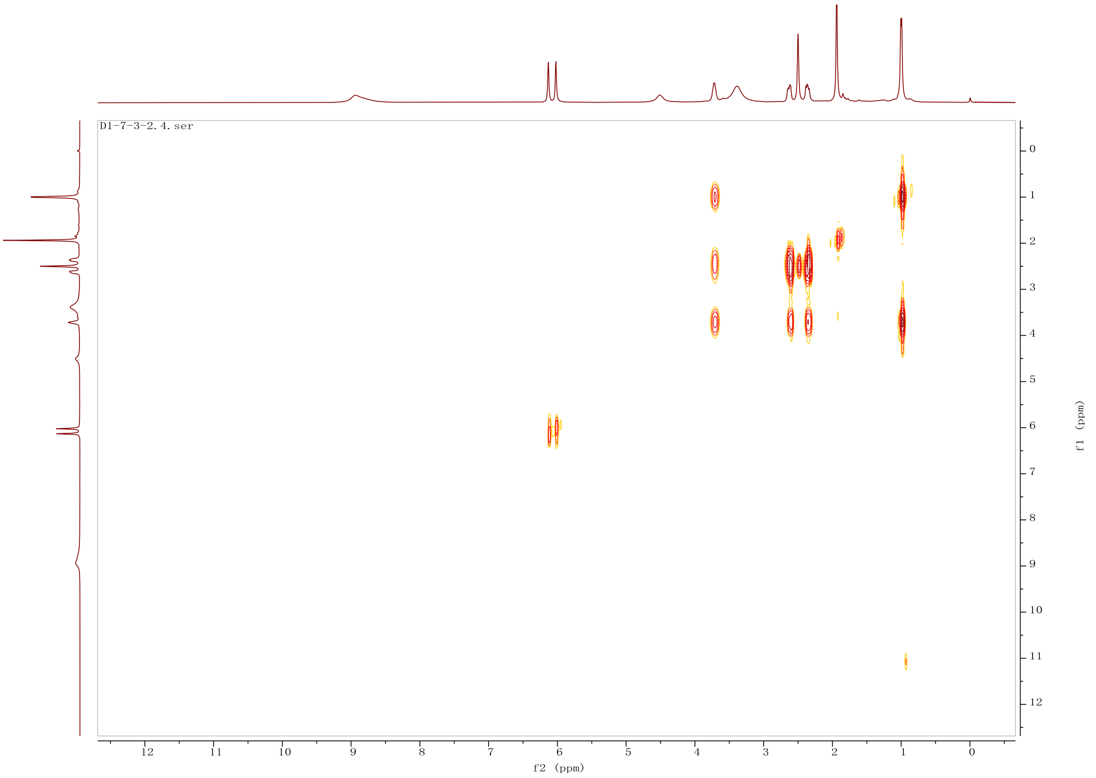

Figure S14 ^{1}H–^{1}H COSY spectra of compound **2** in DMSO-d_6.

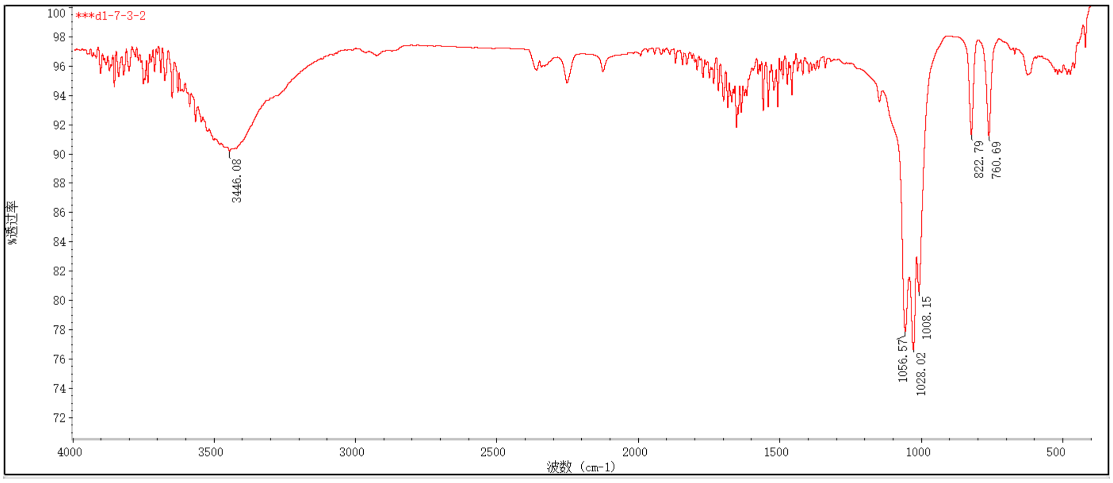

Figure S15 IR data of **2** in KBr

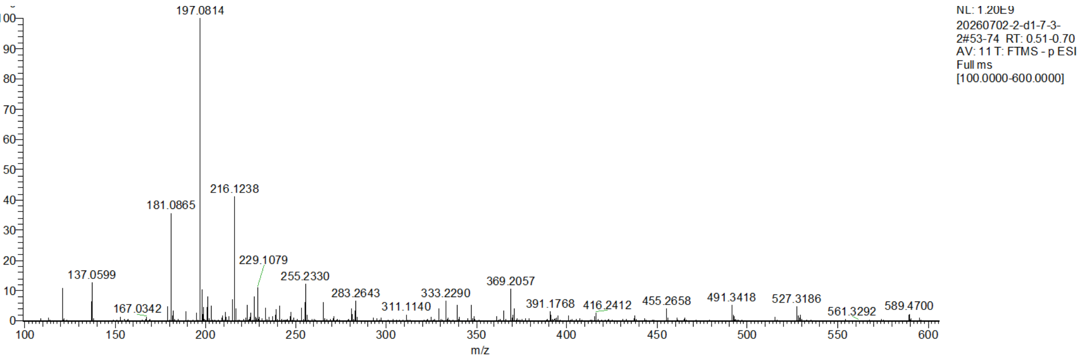

Figure S16 (-)-HR-ESI-MS spectrum of compound **2**.

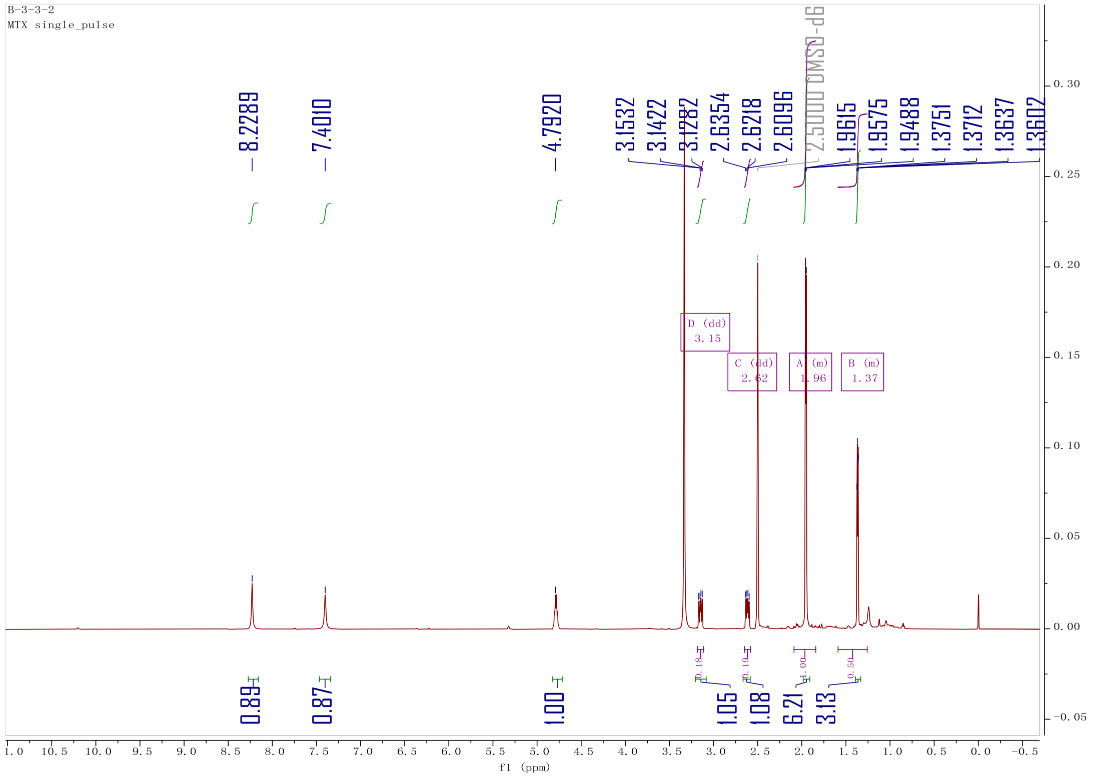

Figure S17 ^{1}H NMR spectrum of compound **3** in DMSO-d_6.

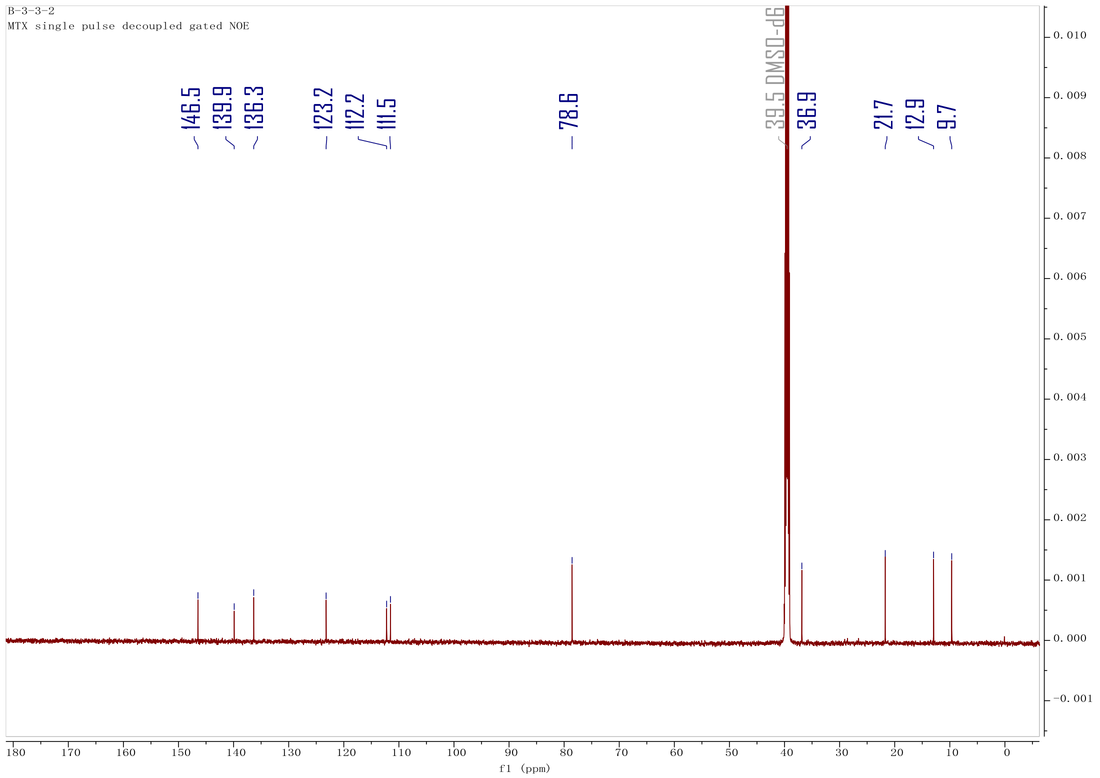

Figure S18 ^{13}C NMR spectrum of compound **3** in DMSO-d_6.

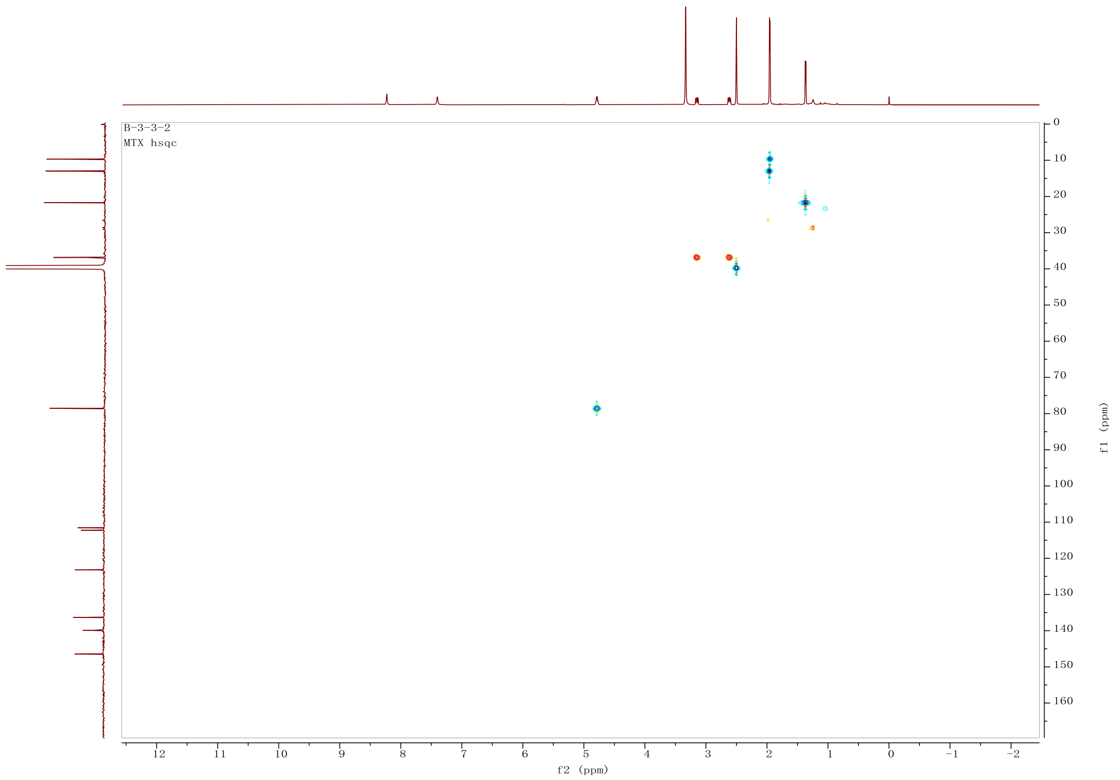

Figure S19 HSQC spectra of compound **3** in DMSO-d_6.

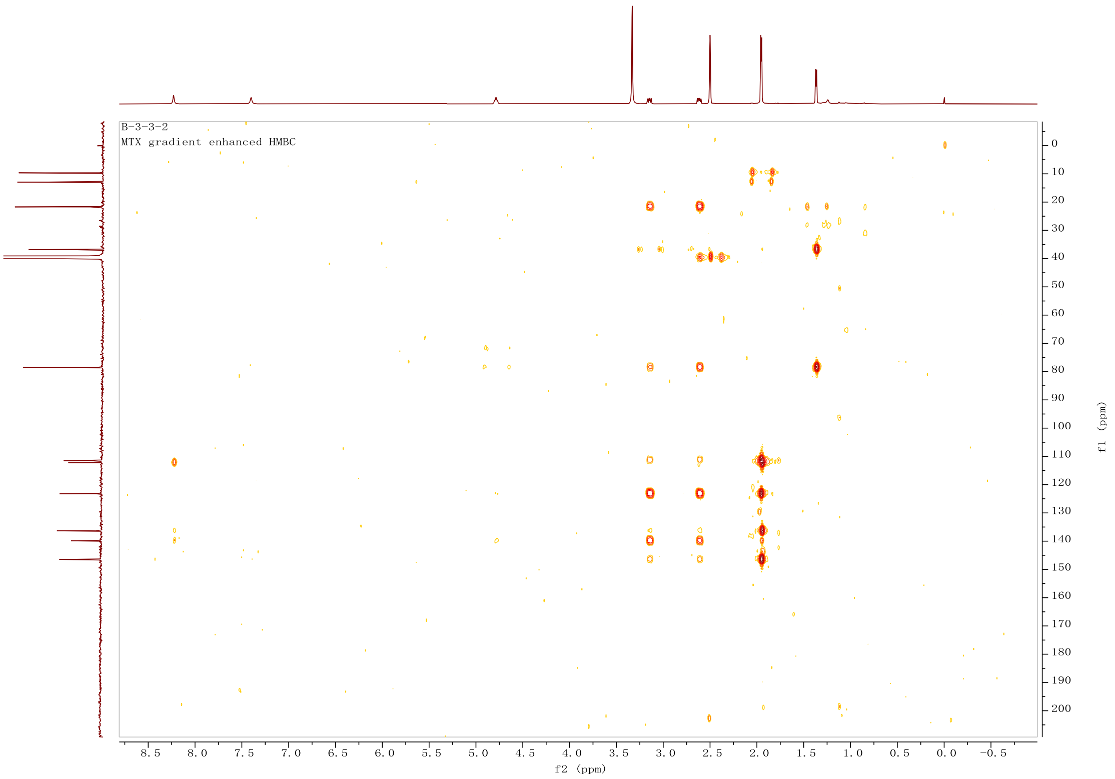

Figure S20 HMBC spectra of compound **3** in DMSO-d_6.

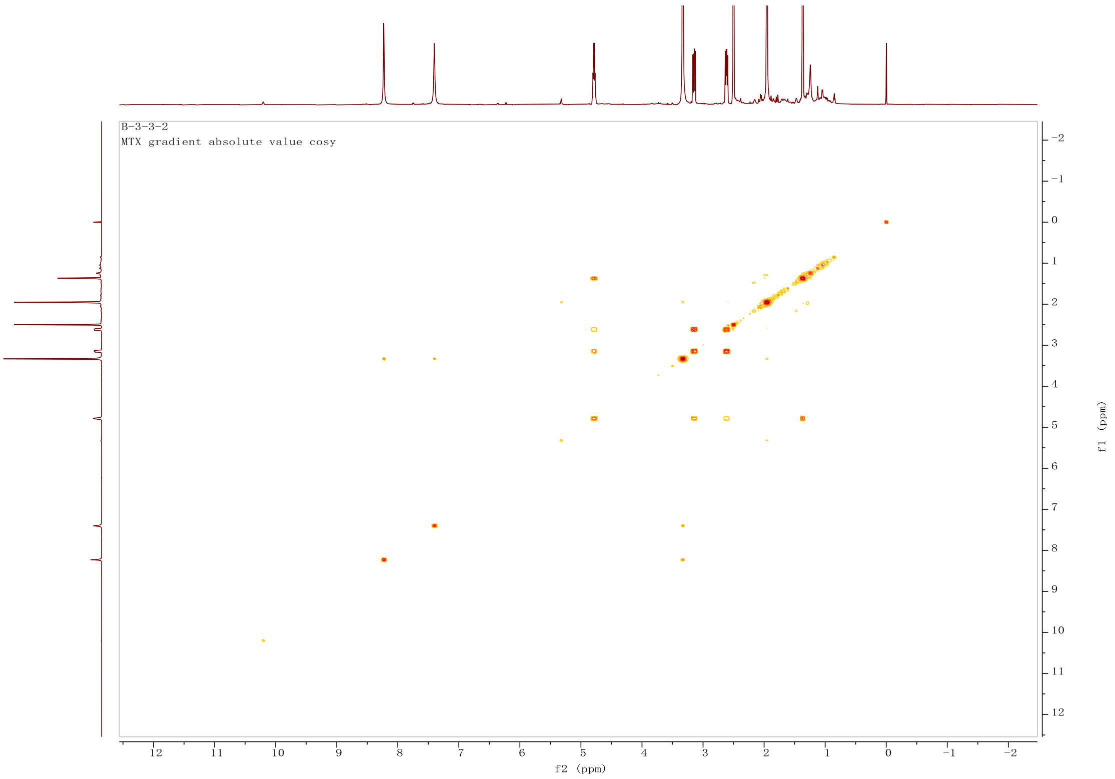

Figure S21 ^{1}H–^{1}H COSY spectra of compound **3** in DMSO-d_6.

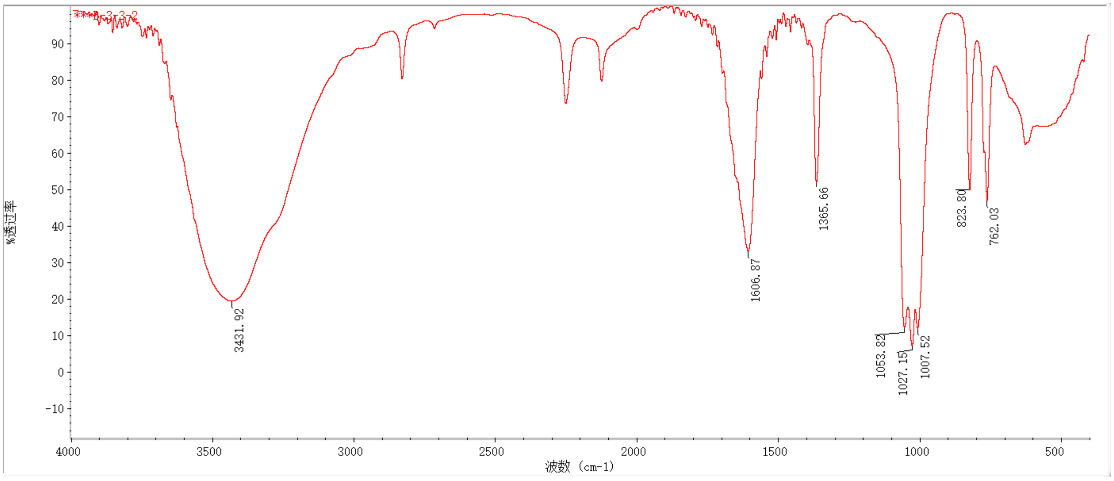

Figure S22 IR data of **3** in KBr

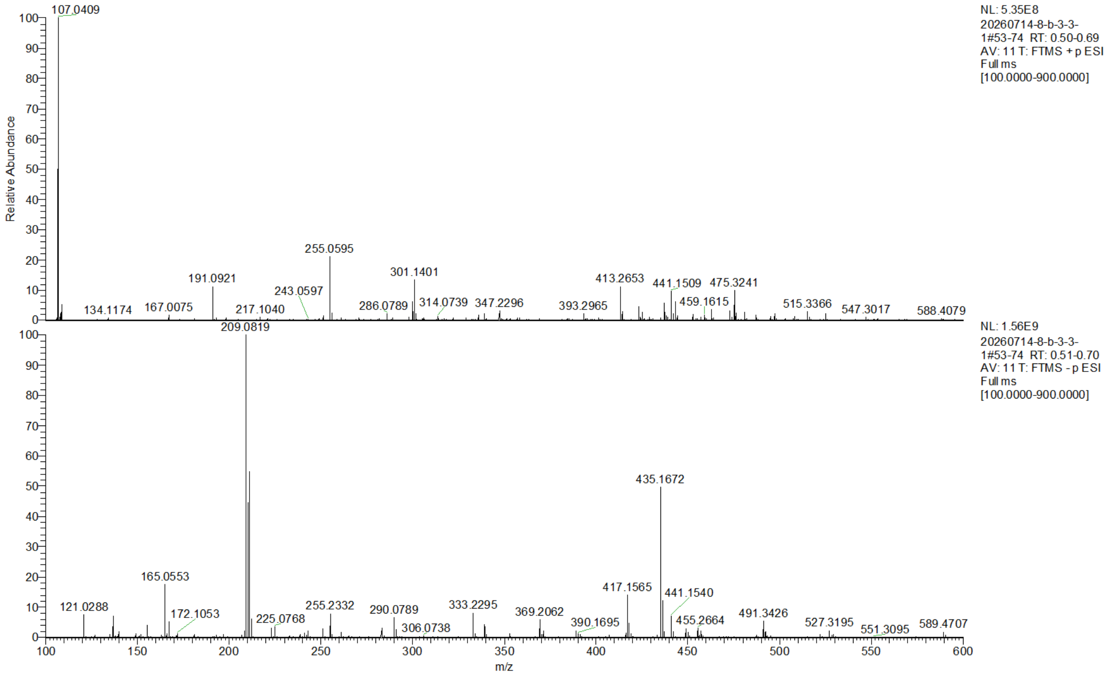

Figure S23 (-)-HR-ESI-MS spectrum of compound **3**.

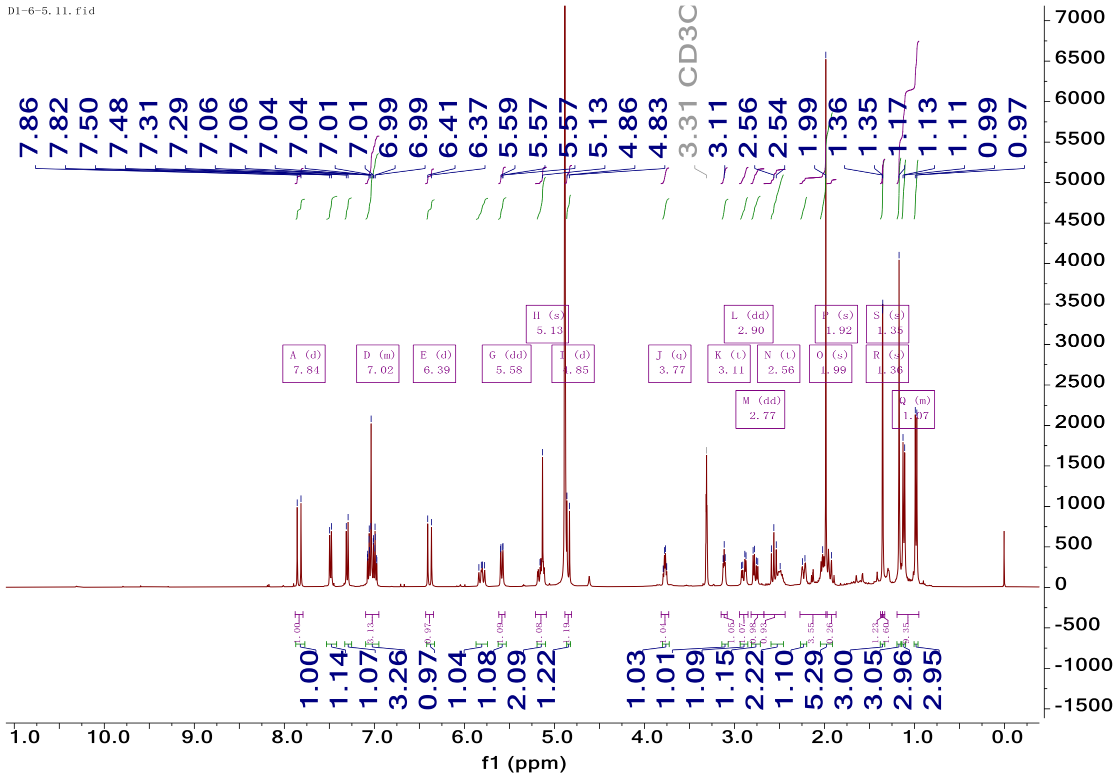

Figure S24 ^{1}H NMR spectrum of compound **5** in CD_3OD.

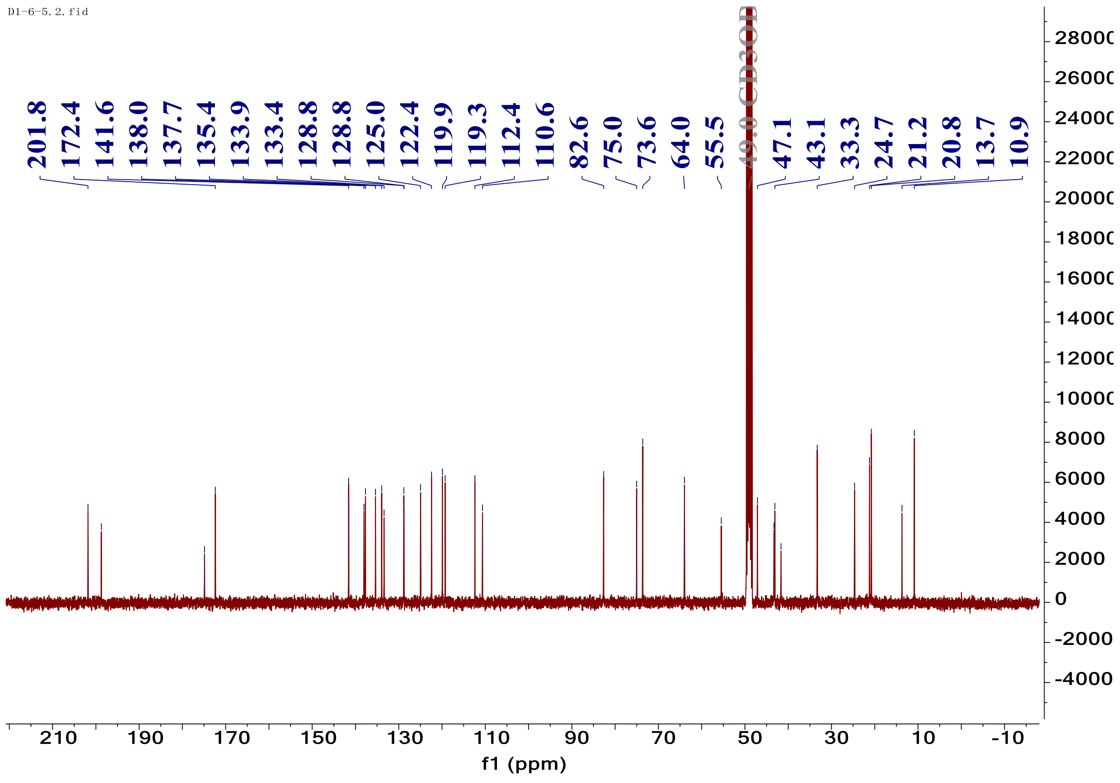

Figure S25 ^{13}C NMR spectrum of compound **5** in CD_3OD.

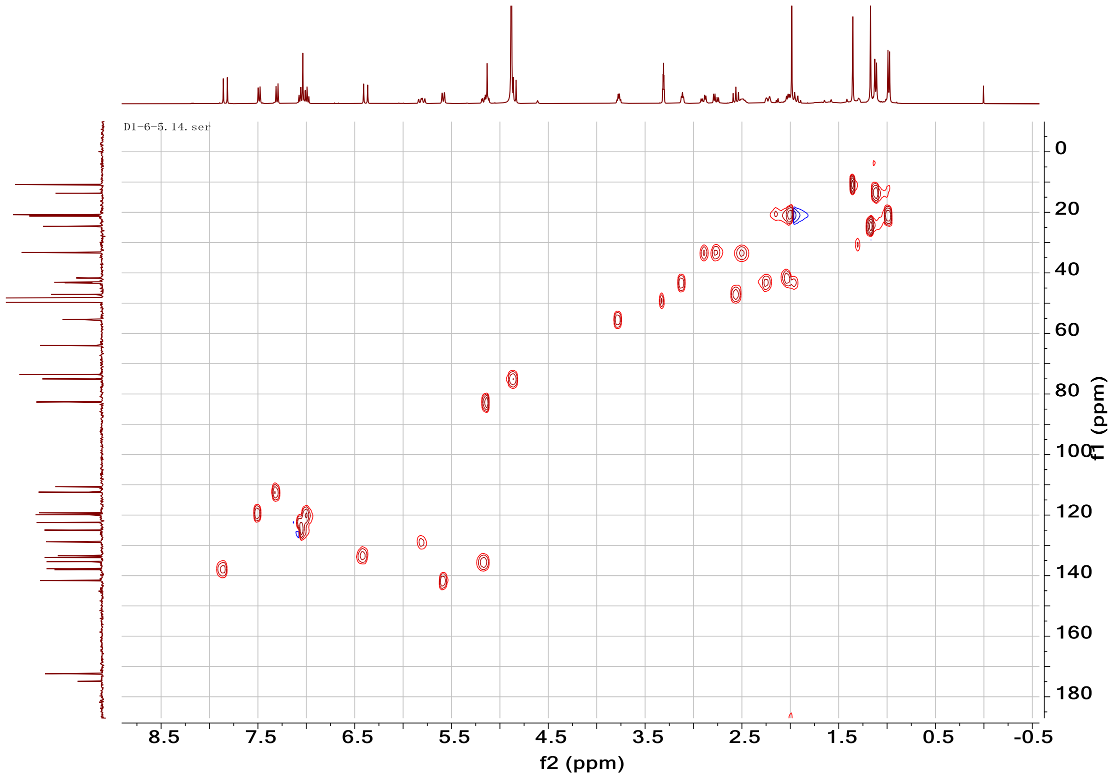

Figure S26 HSQC spectra of compound **5** in CD_3OD.

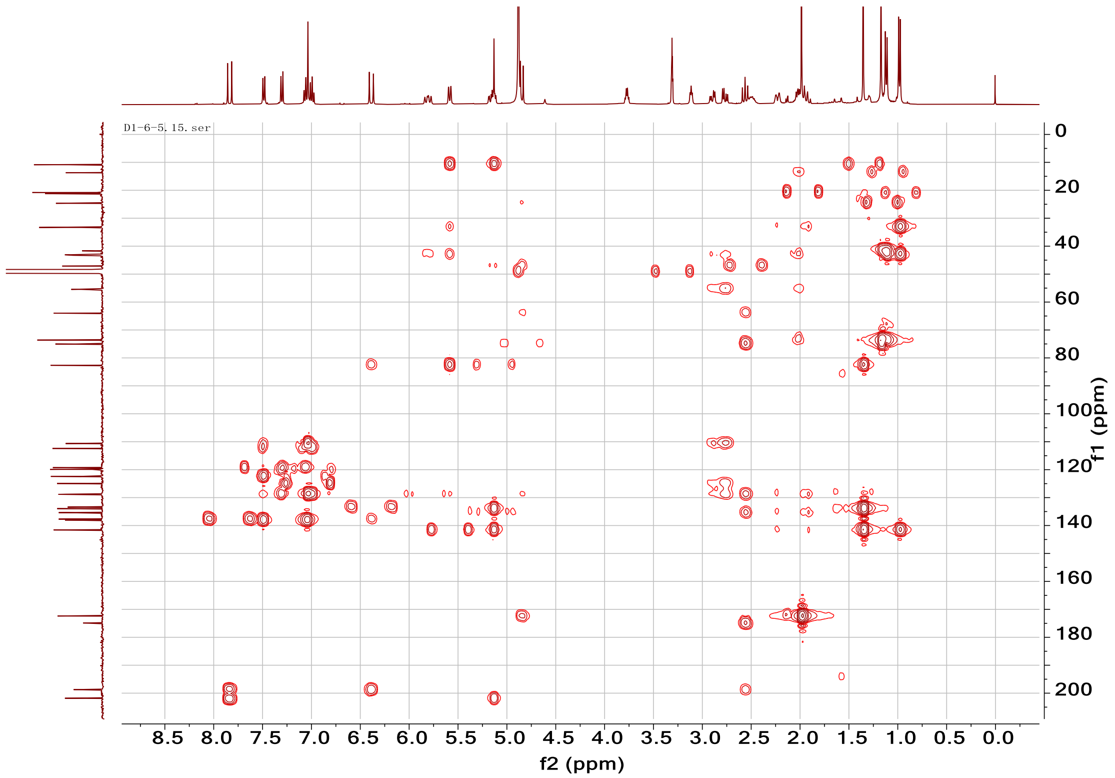

Figure S27 HMBC spectra of compound **5** in CD_3OD.

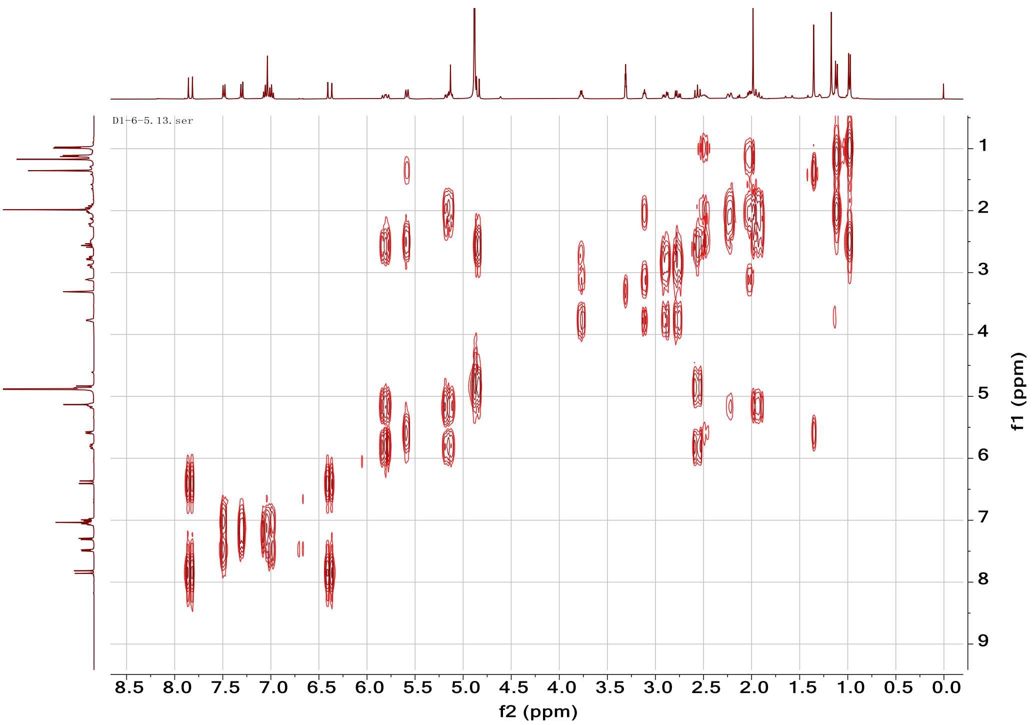

Figure S28 ^{1}H–^{1}H COSY spectra of compound **5** in CD_3OD.

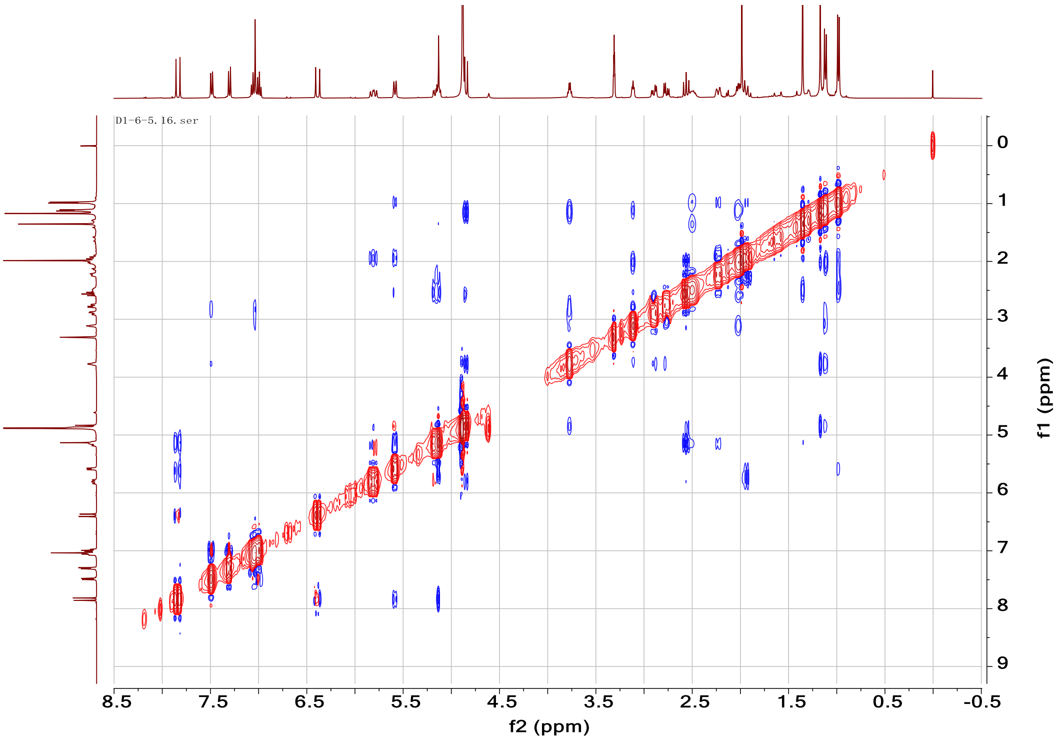

Figure S29 NOESY spectra of compound **5** in CD_3OD.

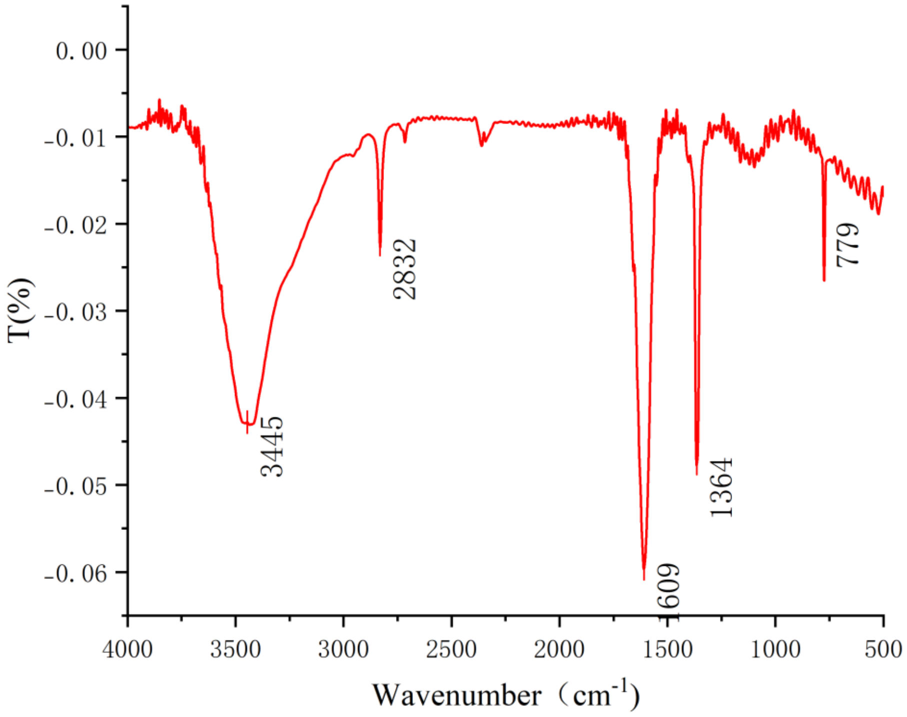

Figure S30 IR data of **5** in KBr

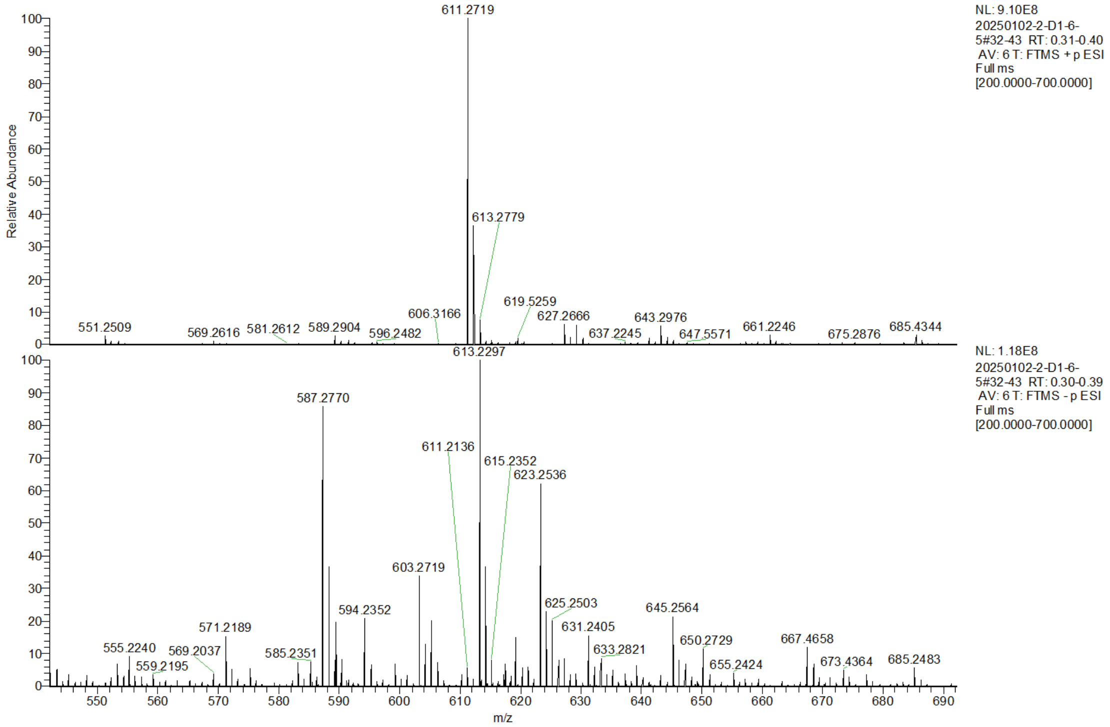

Figure S31 (+)-HR-ESI-MS spectrum of compound **5**.

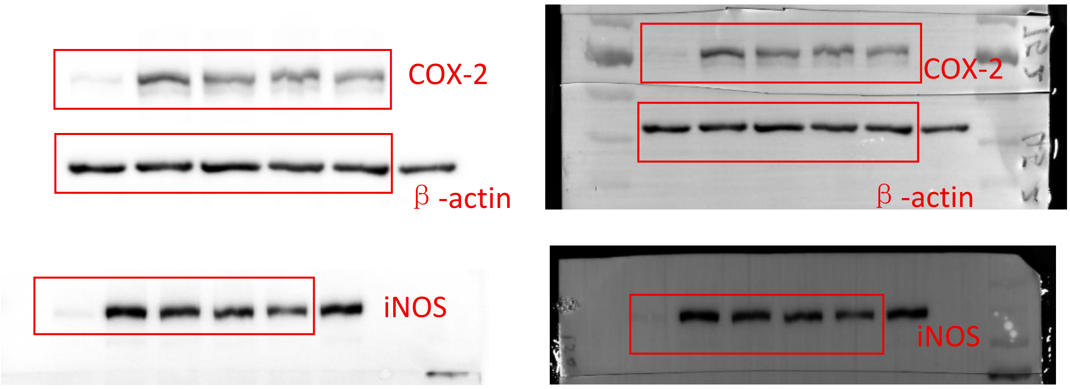

Figure S32 Original, uncropped Western blot images corresponding to Figure 6 D.

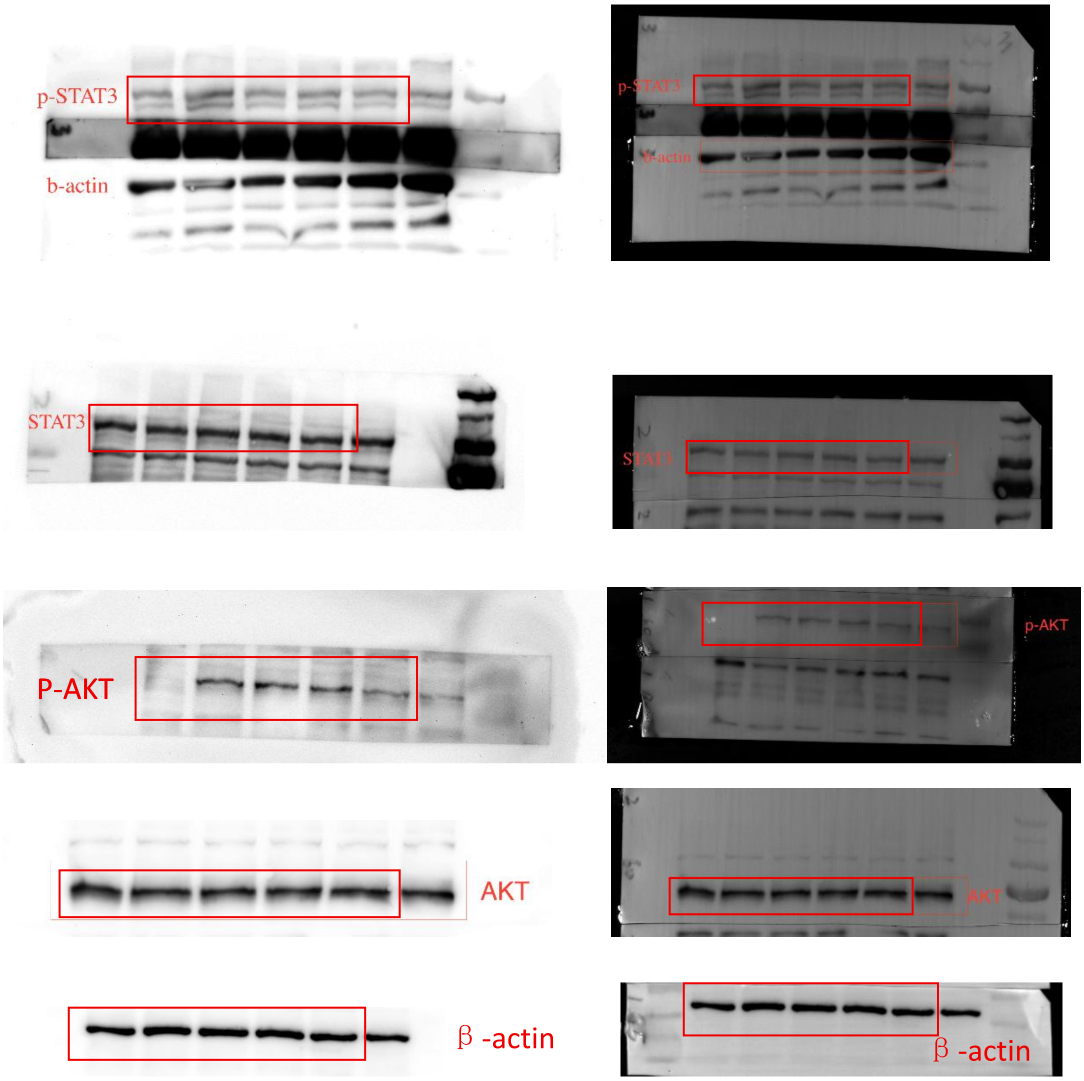

Figure S33 Original, uncropped Western blot images corresponding to Figure 8 A.
